# Highly Efficient Deep Blue Luminescence of 2-Coordinate Coinage Metal Complexes Bearing Bulky NHC Benzimidazolyl Carbene

**DOI:** 10.3389/fchem.2020.00401

**Published:** 2020-05-08

**Authors:** Rasha Hamze, Muazzam Idris, Daniel Sylvinson Muthiah Ravinson, Moon Chul Jung, Ralf Haiges, Peter I. Djurovich, Mark E. Thompson

**Affiliations:** Department of Chemistry, University of Southern California, Los Angeles, CA, United States

**Keywords:** OLED, TADF, high efficiency, deep blue, copper, silver, gold, short lifetime

## Abstract

The structural, photophysical and electrochemical properties of three luminescent 2-coordinate coinage metal (i.e., M = Cu, Ag, Au) complexes bearing a sterically bulky benzimidazolyl carbene, 1,3-bis(2,6-diisopropylphenyl)-1-H-benzo[d]imidazol-2-ylidene (**BZI**), and carbazolide (Cz) as the anionic ligand were investigated. All the complexes emit in the deep blue region (~430 nm) with relatively narrow spectra (full width at half maximum = 44 nm, 2,300 cm^−1^) characterized by vibronic fine structure in nonpolar media (methylcyclohexane at room temperature), and with high photoluminescence quantum yields (Φ_PL_ > 80%) and radiative rate constants (*k*_*r*_ ~ 7.8 × 10^5^ s^−1^). The luminescence is solvatochromic, undergoing a red-shift in a polar solvent (CH_2_Cl_2_) at room temperature that are accompanied by a decrease in quantum yields (Φ_PL_ < 23%) and radiative rate constants (*k*_*r*_ < 4.0 × 10^4^ s^−1^), whereas the non-radiative rate constants remain nearly constant (*k*_*nr*_ ~ 1.0 × 10^5^ s^−1^). The radiative rate is controlled via thermally assisted delayed fluorescence (TADF) and temperature-dependent luminescence studies of the gold complex (**Au**^BZI^) in methylcyclohexane solution reveal an energy difference between the lowest singlet and triplet excited states of 920 cm^−1^. An organic light-emitting diode (OLED) fabricated using **Au**^BZI^ as a luminescent dopant has an external quantum efficiency of 12% and narrow, deep-blue emission (CIE = 0.16, 0.06).

## Introduction

**Figure 1:**
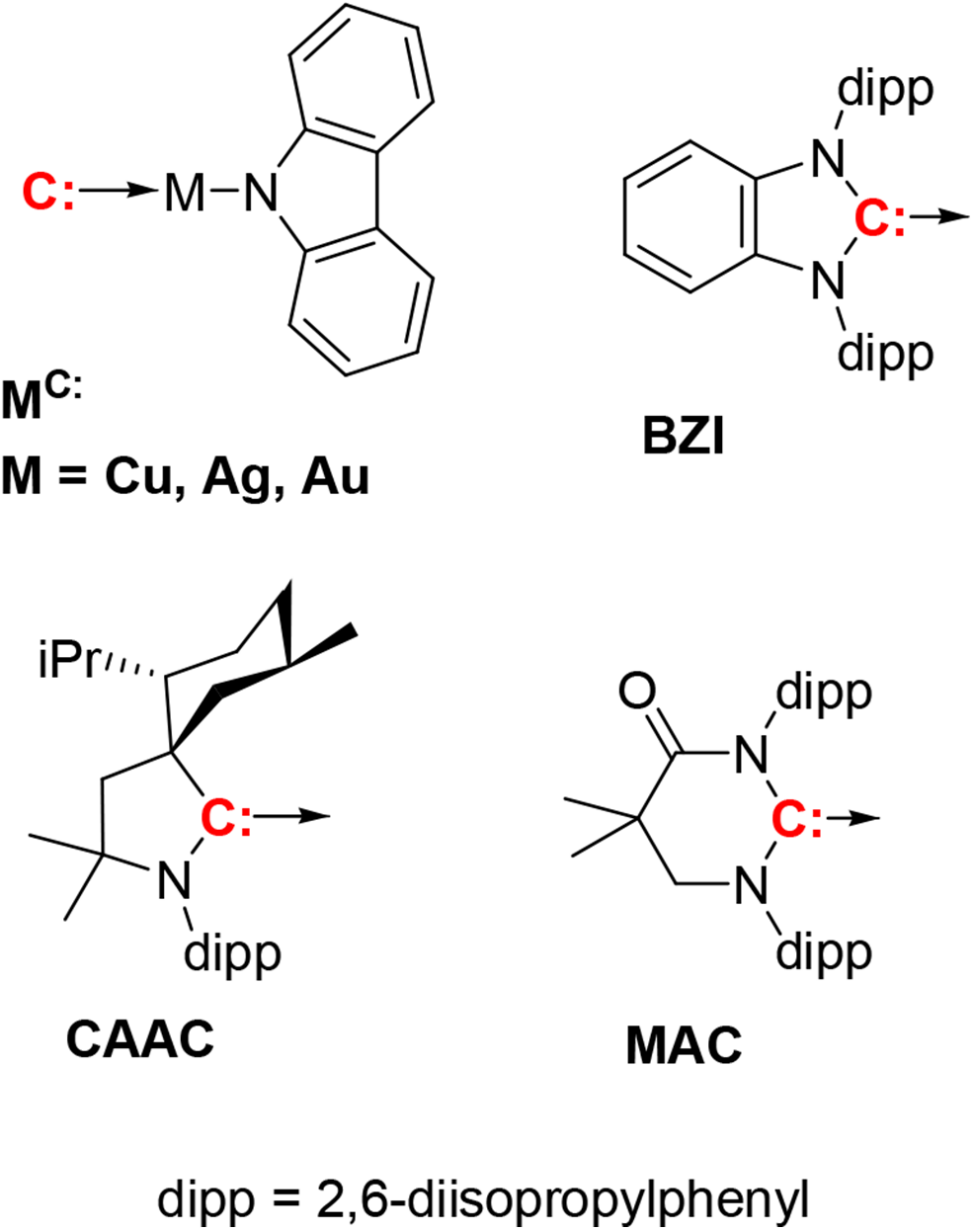
The compounds discussed in this paper have the general formula (carbene)M(N-carbazolyl), with three carbenes, i.e., CAAC, MAC, and **BZI**. The **BZI** compounds are new and the CAAC and MAC derivatives have been reported previously (Hamze et al., 2019a,b; Shi et al., 2019). Compounds are abbreviated using the symbol for the metal ion and superscripted carbene, e.g., **Cu**^CAAC^ or **Au**^BZI^.

Highly luminescent, neutral two-coordinate, linear d^10^ metal complexes of coinage metals i.e., Cu(I), Ag(I), Au(I) have recently been reported (Di et al., 2017; Romanov et al., 2018, 2019; Hamze et al., 2019a,b; Shi et al., 2019). These complexes have redox active carbene (acceptor) and amide (donor) ligands connected by a metal in a linear fashion i.e., (carbene)M^(I)^(amide). Their photoluminescence efficiencies are close to 100% in solution and in thin films, with phosphorescent lifetimes in the range of 1–3 μs. These (carbene)M^(I)^(amide) complexes emit from an amide-N (donor) to carbene-C (acceptor) intramolecular charge transfer (ICT) state, a transition also referred to as ligand-to-ligand charge transfer (LLCT). Because of their excellent photophysical properties, these complexes are potential candidates for application in photocatalysis (Kalyanasundaram, 1982), chemo- and biosensing (Keefe et al., 2000; Lo et al., 2010), dye-sensitized solar cells (Grätzel, 2005) and organic electronics (Lamansky et al., 2001). In particular, their fast radiative lifetimes make them promising candidates as dopants in organic light emitting diodes (OLEDs) (Di et al., 2017; Romanov et al., 2018, 2019; Hamze et al., 2019a,b; Shi et al., 2019). Unlike phosphors like iridium and platinum complexes, which rely on strong spin-orbit coupling (SOC) to induce what is principally triplet metal ligand charge transfer (^3^MLCT) emission (Yersin et al., 2011), the two-coordinate coinage metal complexes emit via E-type fluorescence or thermally activated delayed fluorescence (TADF). Their fast radiative lifetimes are due to two factors; the small energy separation between their lowest singlet and triplet excited states (Δ*E*_ST_) and spin orbit coupling via the metal ion. Together these two parameters lead to rapid endothermic intersystem crossing from the long-lived triplet to the faster radiating singlet state. Recent work has shown that Δ*E*_ST_ in these complexes is dependent on the identity of the metal atom. The copper and gold complexes have similar values for Δ*E*_ST_, whereas the Δ*E*_ST_ for the silver complexes is smaller (Romanov et al., 2018; Hamze et al., 2019b). The small Δ*E*_ST_ in the silver analogs leads to extremely fast radiative rate constants (*k*_*r*_ > 10^6^ s^−1^), faster than the Ir and Pt phosphorescent emitters. The emission energy of complexes, although nearly independent of the metal, can be altered using different carbenes with varying electrophilicity or amides with different nucleophilicity, allowing the luminescence color to be varied from deep blue to deep red (Hamze et al., 2019a; Shi et al., 2019). Developing alternative blue dopants is crucial in tackling the long-standing problem of stability in blue OLEDs. Complexes based on the CAAC ligand (**M**^CAAC^, Figure 1) are reported to have efficient blue photoluminescence and give good efficiencies as dopants in OLEDs (Hamze et al., 2019a). Unfortunately, the emission spectra of these complexes are broad, which is not ideal for display applications. Additionally, the ability to vary the physical and electronic properties of CAAC ligands is limited and therefore inconvenient for modifying the characteristics needed for OLEDs. Complexes based on the MAC ligand (**M**^MAC^) can be used to fabricate high efficiency OLEDs; however, emission in these derivatives is bathochromically shifted to green owing to the electrophilic MAC ligand (Hamze et al., 2019b). Substitution of the carbazole ligand with cyano groups was therefore used to stabilize the HOMO and blue-shift the emission (Shi et al., 2019). Alternatively, carbenes based on benzoimidazoles (BZI), originally used in luminescent two-coordinate Au complexes (Wang et al., 1999), lead to metal complexes with LUMO energies similar to CAAC (Krylova et al., 2014; Hamze et al., 2017, 2020), suggesting that replacing CAAC with benzoimidazolyl-carbene ligands should give similar photophysical and electrochemical properties as **M**^CAAC^. Herein, we examine monovalent, linear, 2-coordinate coinage metal (i.e., M = Cu, Ag, Au) complexes (**M**^BZI^) bearing a sterically bulky benzimidazolyl carbene, 1,3-bis(2,6-diisopropylphenyl)-1-H-benzo[d]imidazol-2-ylidene (**BZI**), and carbazolide (Cz) as the anionic ligand. We have investigated the structural and photophysical properties of the **M**^BZI^ derivatives to elucidate the role of the carbene and the metal ion in the excited-state properties. The **M**^BZI^ complexes have structures, redox potentials and photoluminescent efficiencies (Φ_PL_ = 0.8–1.0) similar to the **M**^CAAC^ analogs, but different excited-state dynamics. Analysis of the luminescence at low temperature reveals that the triplet carbazole (^3^Cz) state and the singlet/triplet intramolecular charge transfer (^1/3^ICT) manifold of the **M**^BZI^ complexes are near degenerate, resulting in photophysical properties that are distinct from the **M**^CAAC^ complexes. The **Au**^BZI^ complex has also been successfully employed a dopant to fabricate efficient blue OLEDs.

## Results and Discussion

### Synthesis

**Figure 2:**
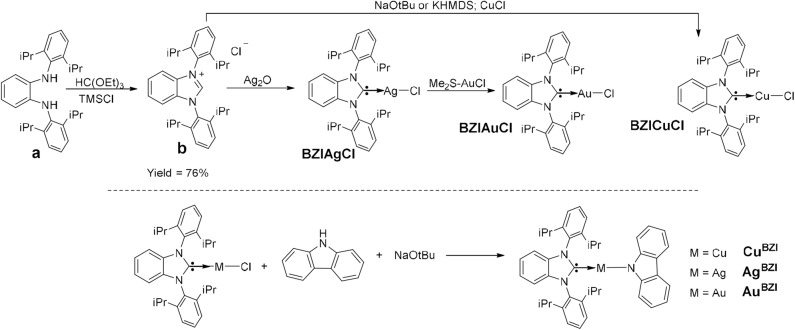
Synthetic route used in the preparation of two-coordinate coinage metal complexes **M**^BZI^.

The synthesis of **M**^BZI^ complexes is depicted in Figure 2. Cyclization of the dianiline derivative (**a)** to the benzoimidazolium salt is reported to be extremely challenging, due to steric bulk imposed by the isopropyl groups, with a reaction yield of only 16% (Grieco et al., 2015). We modified the literature procedure, using excess triethyl orthoformate [HC(OEt)_3_] and distilling the excess off during the reaction, which increases the yield of the reaction to 76%. Similar to **M**^CAAC^ and **M**^MAC^ complexes, the synthesis of **M**^BZI^ complexes starts with addition of Ag_2_O to the benzoimidazolium salt or CuCl to BZI carbene generated *in situ* with base to form the respective benzoimidazole silver(I) or copper(I) chloride complexes. The isolated BZIAgCl is transmetallated with (Me_2_S)AuCl to form the BZIAuCl complex. Reaction of the chloride complexes with carbazole in the presence of NaOtBu forms the **M**^BZI^ complexes in good yields (70–85%).

### X-Ray Analysis

**Figure 3:**
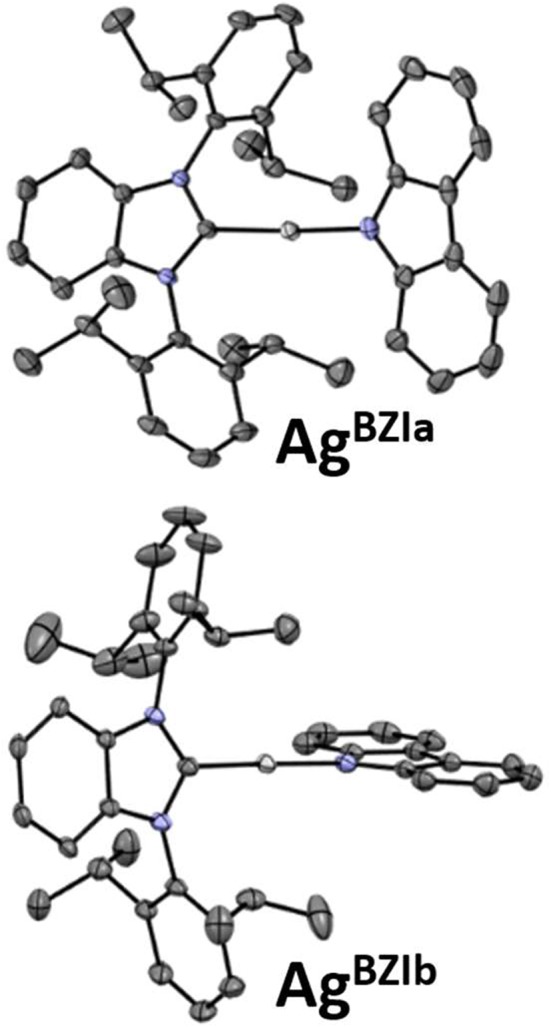
Thermal ellipsoid plots for the conformers of **Ag**^BZIa^ and **Ag**^BZIb^.

The structures were determined for **Cu**^BZI^, **Ag**^BZI^, and **Au**^BZI^ by single crystal X-ray diffraction (details can be found in the Supplementary Information and the datasets generated for this study can be found in the Cambridge Crystallographic Data Center, https://www.ccdc.cam.ac.uk/structures/, under the identifiers **Cu**^BZI^: 1984269, **Ag**^BZI^: 1984268 and **Au**^BZI^: 1984267). **Cu**^BZI^ and **Au**^BZI^ show only a single conformer, with bond distances and interligand torsion angles similar to our previously reported linear coinage metal complexes (summarized in the Supporting Information) (Di et al., 2017; Romanov et al., 2018, 2019; Hamze et al., 2019a,b; Shi et al., 2019). In contrast, the unit cell for the **Ag**^BZI^ complex contains two conformers (Figure 3). The first, like its copper and gold analogs, has a coplanar conformation of its carbene and amide ligands (dihedral angle = 0°), whereas the second displays an orthogonal conformation (dihedral angle = 95°). The C_carbene_···N_Cz_ distances of **M**^BZI^ fall in the order Cu (~3.73 Å) < Au (~4.00 Å) < Ag (~4.11 Å). The C–M–N bond angles are all close to 180° (range = 174–180°).

### Electrochemistry

The electrochemical properties of the complexes were determined using cyclic voltammetry (CV) and differential pulse voltammetry (DPV). The copper and the silver complexes show irreversible reduction, whereas the gold analog shows a quasi-reversible reduction (see section Supporting Information). The reduction potentials for the **M**^BZI^ series are identical (*E*_red_ = −2.84 ± 0.02 V) and greater (more negative) than those of **M**^CAAC^ (*E*_red_ = −2.78 ± 0.06 V) and **M**^MAC^ (*E*_red_ = −2.45 ± 0.06 V) complexes. The reduction potentials of **M**^BZI^ relative to their **M**^CAAC^ and **M**^MAC^ analogs indicates that the electrophilicity of the coordinated BZI carbene is lower than the CAAC and MAC ligands in similar complexes. All the complexes undergo irreversible oxidation (Table 1) and, unlike the **M**^CAAC^ and **M**^MAC^ complexes where the oxidation potential is the same across the series, the potential of the **M**^BZI^ complexes increases from Cu (*E*_ox_ = 0.11 ± 0.06 V) to Au (*E*_ox_ = 0.32 ± 0.06 V), suggesting participation of the metal in the oxidation process. Thus, values for the redox gap (Δ*E*_redox_) are greater for the silver and gold complexes than the copper analog.

**Table 1:** Redox potentials of complexes **M**^BZI^ and the associated experimental frontier orbital energies.

**Complex**	***E*_**ox**_ (V)**	***E*_**red**_ (V)**	**Δ*E*_**redox**_ (V)**	***E*_**HOMO**_ (eV)**	***E*_**LUMO**_ (eV)**
**Cu**^BZI^	0.113	−2.85	2.96	−4.92	−1.47
**Ag**^BZI^	0.284	−2.84	3.12	−5.12	−1.48
**Au**^BZI^	0.318	−2.82	3.14	−5.16	−1.51

*Electrochemical studies performed in acetonitrile with tetrabutylammonium hexafluorophosphate (TBAF) as the electrolyte and referenced to Fc^+^/Fc. The redox values were obtained using DPV and converted to HOMO/LUMO energies using the equations in Sworakowski et al. (2016)*.

### Computational Analysis

**Figure 4:**
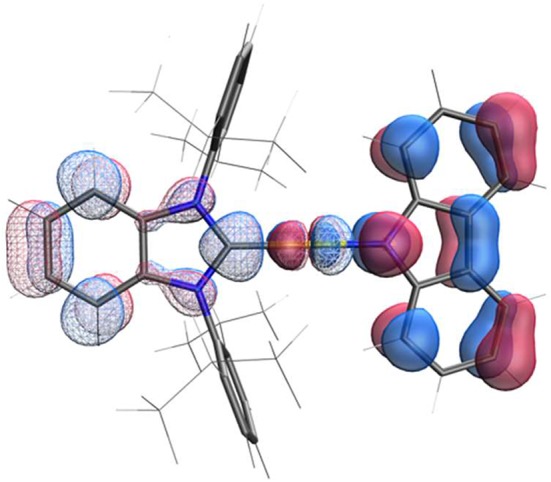
HOMO (*E* = 4.22 eV, solid) and LUMO (*E* = 1.44 eV, mesh) surfaces of complex **Au**^BZI^.

The structure calculated using density function theory (DFT) for the ground state of **Au**^BZI^ is shown in Figure 4. The HOMO density is localized largely on the carbazolide (Cz) ligand, whereas the LUMO is primarily confined to the carbene ligand, with smaller contributions from the metal d-orbitals to both MOs. Time dependent DFT (TD-DFT) calculations find that ^3^Cz is the lowest-energy state and lies <0.09 eV below the manifold of the ^1/3^ICT states (Table S8). Additionally, the oscillator strength calculated for the silver complex is weaker than that of its Cu and Au analogs (Table S8), consistent with the lower molar absorptivity observed for the ICT transition in the absorption spectra of the silver complex (see below). Large molecular dipole moments calculated for the ground state are directed along the metal-ligand bond axis toward the carbazolide ligand (μ_calc_ ≈ −12.5 debye), whereas the moments for the excited ^1^ICT state is comparable in magnitude but directed toward the BZI ligand (μ_calc_ ≈ 13.5 debye).

### Photophysical Characterization

**Figure 5:**
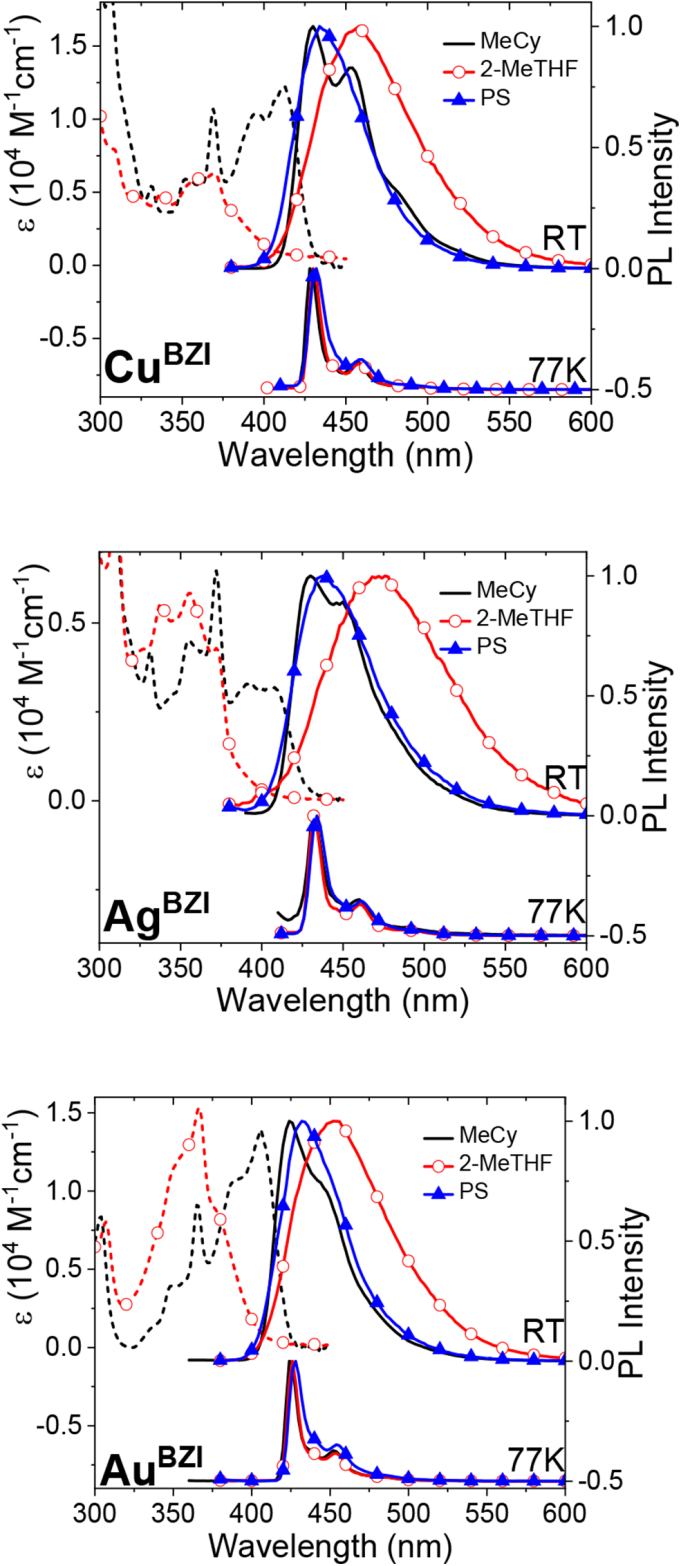
Absorption (dashed lines) and photoluminescence (solid line) spectra of **M**^BZI^ complexes in MeCy, 2-MeTHF and polystyrene at room temperature (RT) and 77 K.

Absorption spectra of the **M**^BZI^ complexes in polar (2-methyltetrahydrofuran, 2-MeTHF) and nonpolar (methylcyclohexane, MeCy) solvents are shown in Figure 5. Absorption bands between 300 and 375 nm of **M**^BZI^ are assigned to π-π^*^ transitions localized on the carbazolyl ligand (Hamze et al., 2019a,b; Shi et al., 2019). The band at lower energy (>375 nm) is assigned to an intramolecular ligand-to-ligand charge transfer (ICT/LLCT) transition from Cz to **BZI**. The energy of the ICT band of **M**^BZI^ is higher than in the **M**^CAAC^ and **M**^MAC^ complexes, consistent with the order of reduction potentials in these complexes. The ICT band extends to 410 nm in MeCy and has two features separated by 1100 cm^−1^ indicative of vibronic coupling. Similar to **M**^CAAC^ and **M**^MAC^ complexes, the ICT band of the **M**^BZI^ complexes displays negative solvatochromism and merges into the higher lying ligand π-π^*^ transitions in polar solvents. These shifts with solvent polarity are due to the large change in the molecular dipole moments between the ground and excited ICT states. The molar absorptivities of the **M**^BZI^ complexes decrease in the order Au > Cu > Ag in all media. The same trend was observed in the **M**^CAAC^ and **M**^MAC^ analogs and attributed to a decrease in the overlap integrals between orbitals on the donor Cz and acceptor ligands mediated by the metal center (Hamze et al., 2019b).

**Figure 6:**
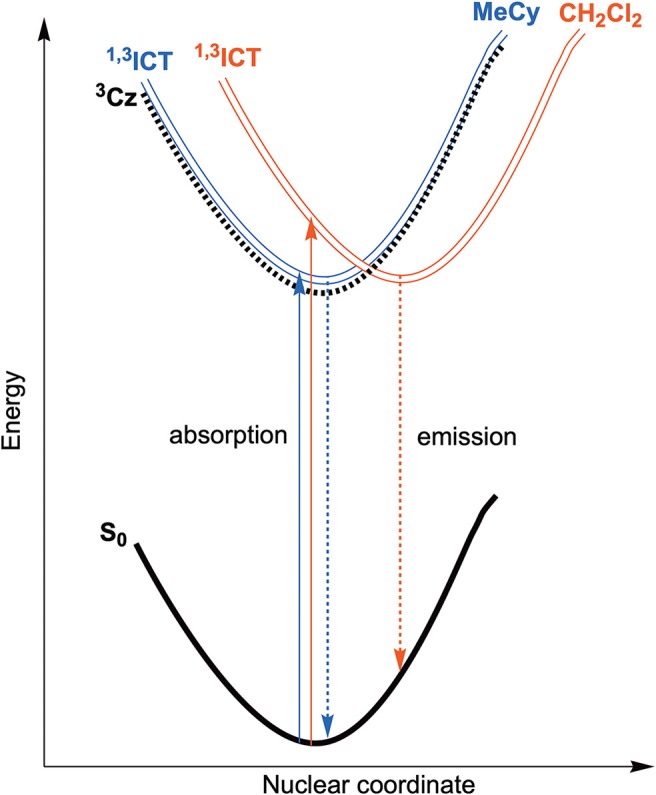
Qualitative energy diagram representing the ground state (S_0_) and both excited state potential energy surfaces (^3^Cz and ^1,3^ICT) as a function of nuclear coordinate in MeCy (blue) and CH_2_Cl_2_ (red) solution along with absorption (solid) and emission (dashed) transitions.

Emission spectra of the **M**^BZI^ compounds in MeCy, 2-MeTHF solution and polystyrene (PS) films at room temperature and 77 K are shown in Figure 5 and tabulated in Table 2. The spectra from the **M**^BZI^ complexes are blue-shifted relative to their **M**^CAAC^ and **M**^MAC^ counterparts, yet display similar solvatochromic behavior, undergoing red shifts in polar solvents. Spectra recorded in MeCy are relatively narrow (FWHM = 44 nm, 2,300 cm^−1^) and show underlying vibronic features. The photoluminescence quantum yields (Φ_PL_) of **M**^BZI^ complexes are close to unity in MeCy and PS films but decrease with increasing solvent polarity (Table 2). Furthermore, increasing solvent polarity is correlated with increased spectral width and loss of the vibronic features, suggesting structural distortion in the excited states. To explain the blue-shift in absorption spectra and red-shift in emission spectra observed in these complexes with increasing solvent polarity, a diagram representing the potential energy surfaces for the ground state (S_0_) and excited states (^3^Cz and ^1,3^ICT) as a function of nuclear coordinates in MeCy and CH_2_Cl_2_ is proposed (Figure 6). The vibronically structured absorption and emission spectra in nonpolar solvents (MeCy) indicate that the potential energy surfaces are well-nested, such that ICT transitions are induced with small reorganization energies. In contrast, the blue-shifted absorption and broad, featureless red-shifted emission observed in polar solvents (CH_2_Cl_2_) indicate that significant reorganization occurs within the metal complex and its surrounding media (to a larger extent) as a result of the large change in dipole moment upon excitation (Δμ_calc_ > 24 debye). Unlike **M**^CAAC^ and **M**^MAC^ complexes, where the radiative rate constant (*k*_*r*_) is fastest for the silver analog (Romanov et al., 2018; Hamze et al., 2019b), **Au**^BZI^ has the fastest *k*_*r*_ in accord with gold having the largest SOC constant.

**Table 2:** Luminescence properties of complexes **M**^BZI^ in various media.

**Complex**		**λ_max_ (nm)**	**Φ_PL_**	**τ (μs)**	***k_*r*_* (10^5^ s^−1^)**	***k_*nr*_* (10^5^ s^−1^)**	**λ_max, 77K_ (nm)**	**τ_77K_ (μs)**
**Cu**^BZI^	MeCy	428	0.80	1.23	6.50	1.63	428	6300
	Toluene	450	0.75	1.50	5.0	1.7	—	—
	2-MeTHF	458	0.35	2.06	1.7	3.2	430	11000
	CH_2_Cl_2_	466	0.03	1.24	0.24	7.8	—	—
	PS film	434	0.86	0.97 (36%); 4.8 (64%)	2.5^a^	0.41^a^	432	3000
**Ag**^BZI^	MeCy	430	0.58	1.04	5.6	4.4	432	18000
	Toluene	458	0.50	3.27	1.5	1.5	—	—
	2-MeTHF	476	0.19	5.66	0.34	1.4	432	20000
	CH_2_Cl_2_	482	0.03	1.64	0.18	5.9	—	—
	PS film	438	0.85	0.69 (26%); 5.1 (74%)	2.2^a^	0.38^a^	434	6600
**Au**^BZI^	MeCy	424	0.89	1.15	7.8	0.9	424	340
	Toluene	448	0.94	1.11	8.5	5.4	—	—
	2-MeTHF	452	0.79	2.63	3.0	0.8	426	640
	CH_2_Cl_2_	458	0.23	5.79	0.40	1.3	—	—
	PS film	432	1.0	0.74 (46%); 3.6 (54%)	4.4^a^	< 0.04^a^	428	190

a *Calculated from the weighted averages of both contributions*.

The biexponential character of the emission decay in PS thin films could be due to the presence of **M**^BZI^ complexes in different conformations, one where the carbene and carbazole are in a coplanar orientation and another where the two ligands are twisted relative to each other. In our previous studies of (CAAC)Cu(carbazole) complexes we found that while the two forms display similar emission spectra the twisted form has a markedly longer excited state lifetime and lower oscillator strength than the coplanar form (Hamze et al., 2019a). In solution the excited **M**^BZI^ can effectively rotate to the coplanar form prior to relaxing to the ground state. However, the rigid PS matrix will prevent the conformers from equilibrating in the excited state and thus they are expected to emit independently with different individual emission lifetimes.

The emission spectra display a pronounced rigidochromic shift upon cooling to 77 K and become extremely narrow and vibronically structured, with luminescence lifetimes in the millisecond regime. Thus, emission at low temperatures is consistent with a triplet transition localized on the carbazolide ligand (^3^Cz). This change in emission properties with temperature is attributed to the close energy separation between the ^3^Cz and ^1/3^ICT manifolds, making the ICT manifold thermally accessible at room temperature, but inaccessible in frozen MeCy and 2-MeTHF at 77 K. The fact that the **M**^BZI^ complexes display ^3^Cz emission in PS (as well as MeCy and 2-MeTHF) at 77 K is different from the behavior observed in **M**^CAAC^ and **M**^MAC^ complexes. Emission from the latter complexes remains broad and featureless in a polystyrene matrix at all temperatures, even down to 4 K (Hamze et al., 2019b). Thus, in the case of the **M**^CAAC^ and **M**^MAC^ complexes, the ^3^ICT state lies below the energy of the ^3^Cz state in PS at all temperatures (Hamze et al., 2019b). However, for the **M**^BZI^ complexes in PS films, it is evident that the lowest excited triplet state is indeed ^3^Cz at all temperatures. This difference suggests that the ^3^Cz and ^3^ICT states in the **M**^BZI^ complexes are near degenerate in energy, and TADF emission occurs via thermal activation from the ^3^Cz to ^1^ICT states, not just within the ICT manifold as in the case of the **M**^CAAC^ and **M**^MAC^ complexes.

**Figure 7:**
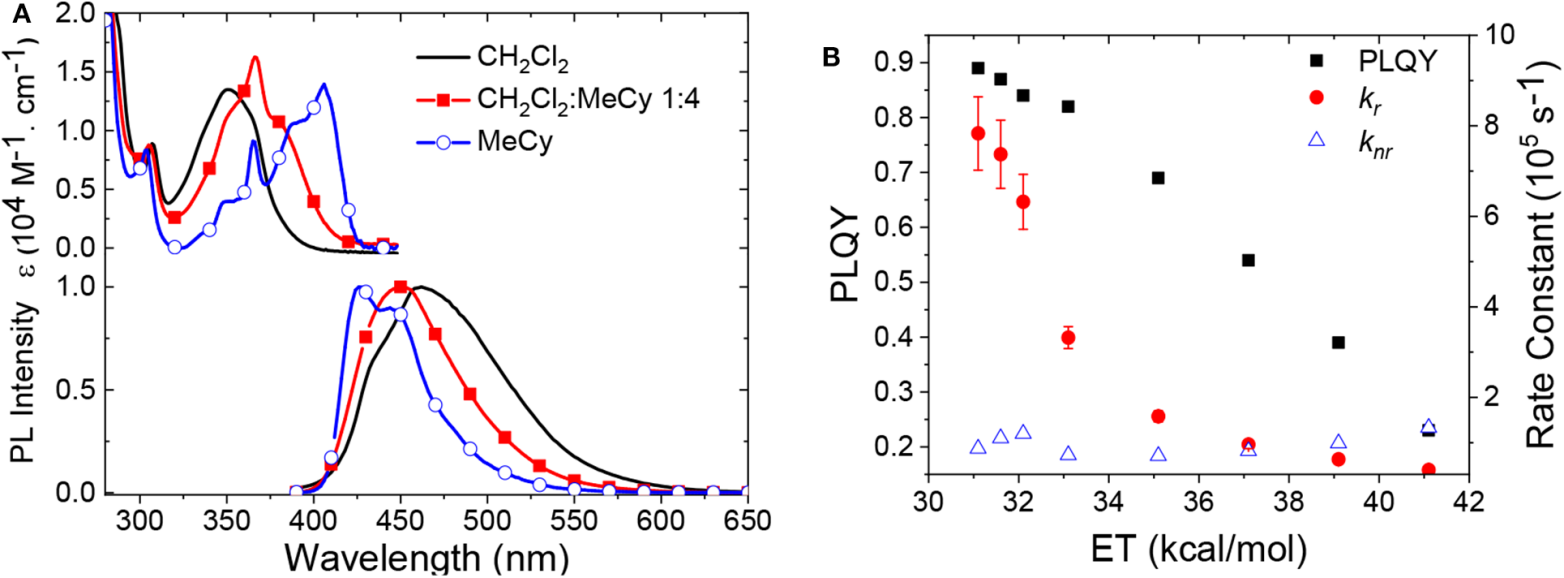
**(A)** Absorption and emission spectra of **Au**^BZI^ in CH_2_Cl_2_, MeCy, and a mixture of both solvents. **(B)** Change in Φ_PL_ and radiative and non-radiative rate constants of **Au**^BZI^ as a function of solvent polarity.

Another difference in the properties of the **M**^BZI^ complexes compared to the **M**^CAAC^ and **M**^MAC^ analogs is the pronounced decrease in luminescence efficiency with increasing solvent polarity. For example, the quantum yield of **Cu**^BZI^ is severely diminished in CH_2_Cl_2_ relative to that recorded in MeCy (Φ_PL_ = 0.03 in the former and 0.80 in the latter), whereas this decrease in efficiency is less pronounced for **Cu**^CAAC^ (Φ_PL_ = 0.4 in CH_2_Cl_2_ and 0.92 in MeCy) and **Cu**^MAC^ (Φ_PL_ = 0.5 in CH_2_Cl_2_ and 0.90 in MeCy). To better understand the origin of this decrease in Φ_PL_ with solvent polarity, photophysical properties of **Au**^BZI^ were characterized in mixtures of MeCy and CH_2_Cl_2_ at various ratios. The ICT band in the absorption spectra gradually blue shifts with increasing CH_2_Cl_2_ concentration and the vibronic fine structure observed in MeCy disappears in mixtures with ≥ 5 vol% CH_2_Cl_2_ (Figure 7A and Figure S6). Figure 7B shows that the radiative rate constant of **Au**^BZI^ decreases with increasing solvent polarity, whereas the non-radiative rate constant (*k*_*nr*_) remains near constant, consequently decreasing the Φ_PL_. The fact that the non-radiative rate constant of **Au**^BZI^ is largely independent of CH_2_Cl_2_ concentration, despite the similar reduction potentials for the **Au**^BZI^ excited state (E^0/+^*^^ = −2.67 V) and CH_2_Cl_2_ (*E*^0/−^ = −2.73 V), indicates that there is no oxidative quenching of excited **Au**^BZI^ by CH_2_Cl_2_. Therefore, the lower Φ_PL_ of **Au**^BZI^ in CH_2_Cl_2_ comes about from a decrease in the radiative rate constant. This change is likely due to a decrease in the Franck-Condon factors for the ICT transition caused by the shift of the excited state surface in the polar solvent, as illustrated in Figure 6.

**Figure 8:**
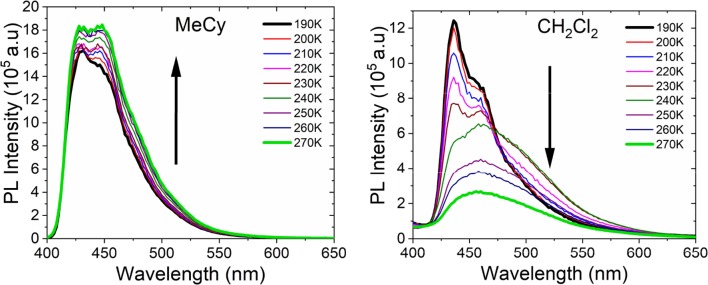
Emission spectra of **Au**^BZI^ in MeCy and CH_2_Cl_2_ at various temperatures.

**Figure 9:**
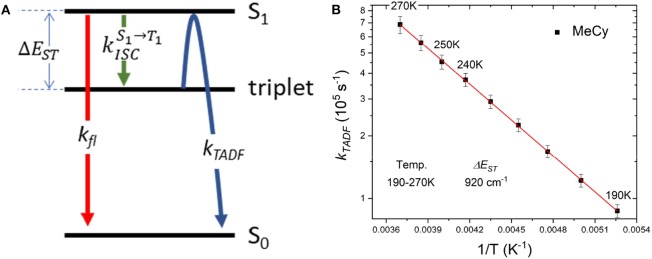
**(A)** Kinetic scheme for emission from **Au**^BZI^. **(B)** Arrhenius plot of the temperature-dependent lifetime data for **Au**^BZI^ in MeCy recorded from 190 to 300 K, along with fits to the data according to Equation 1.

Emission studies of **Au**^BZI^ at variable temperature were conducted in MeCy and CH_2_Cl_2_ to investigate the parameters controlling TADF (Figure 8). Emission in MeCy slightly increases in intensity and displays limited changes in line shape with increasing temperature. In contrast, spectra recorded in CH_2_Cl_2_ reveal a drop in intensity with increasing temperature. Vibronic features resolved at low temperatures (190–230 K) are found to broaden abruptly at 240 K. The *k*_*r*_values calculated from the quantum yields measured at various temperatures (see Supplementary Information) were fit to a two level model using Equation 1 (Figure 9A) (Hamze et al., 2019b).

(1) $$\begin{array}{l} ln\left(k_{TADF}\right)=ln\left(\frac{k_{ISC}^{S_{1}\to T_{1}}}{3}\left(1-\frac{k_{ISC}^{S_{1}\to T_{1}}}{k_{fl}+k_{ISC}^{S_{1}\to T_{1}}}\right)\right)-\frac{{\Delta E}_{ST}}{k_{B}T} \end{array}$$

Where, *k*_*B*_ is the Boltzmann constant, *k*_*TADF*_ and *k*_*fl*_ are the radiative rate constants of the TADF and fluorescence, respectively and $k_{ISC}^{S_{1}\to T_{1}}$ is the intersystem crossing rate (see Figure 9A). The best fit to the MeCy data (Figure 9B) give an energy difference between the triplet and emitting singlet state of **Au**^BZI^ of 920 cm^−1^. However, the Arrhenius plot of the radiative rate constant of **Au**^BZI^ recorded in CH_2_Cl_2_ at variable temperature is decidedly non-linear (Figure S10). Variable temperature NMR spectra indicate that an aquo species is formed with residual water below 240 K (Figure S11). The formation of this aquo complex likely leads to the anomalous behavior observed in CH_2_Cl_2_ at lower temperatures, making the analysis using the simple two-level model problematic.

### OLED Characterization

**Figure 10:**
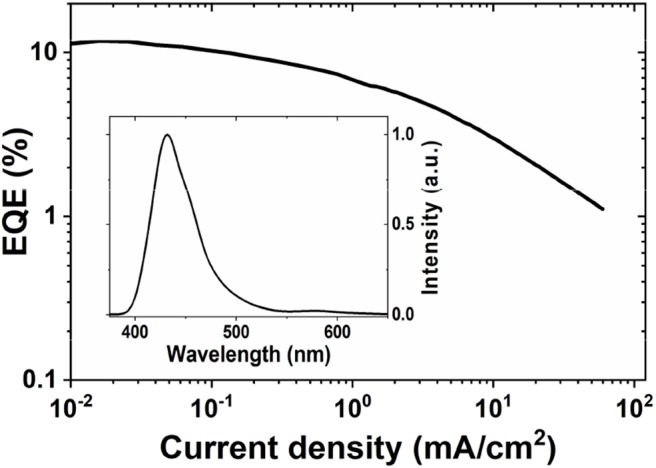
EQE vs. current density curve of 5 vol% doped devices of **Au**^BZI^. The EL spectrum is shown in the inset. Device architecture: ITO/10 nm HATCN/40 nm TAPC/40 nm UGH3:5 wt % **Au**^BZI^ /40 nm TPBI/1 nm LiF/100 nm Al.

The **Au**^BZI^ complex is stable to sublimation and was thus used as dopant to fabricate OLEDs by thermal evaporation. Device optimization and details are shown in the Supplementary Information. Considering the high triplet energy of **Au**^BZI^ (E_T_ = 3.1 eV), 1,3-bis(triphenylsilyl)benzene (UGH3, E_T_ = 3.5 eV) was employed as the host matrix. Devices were fabricated using different doping levels (5, 10 and 15 wt%) and the best performance was obtained with 5 wt% **Au**^BZI^ (see Supplementary Information). Optimized devices achieved reasonably high efficiencies (maximum EQE = 12%, Figure 10) and electroluminescence (EL) spectra (λ_max_ = 430 nm, FWHM = 45 nm) identical to the PL spectrum in PS, demonstrating efficient exciton confinement on the complex. The color coordinates of the EL spectrum (CIE = 0.16, 0.06) make **Au**^BZI^ an efficient *deep blue* dopant for phosphorescent OLEDs.

## Conclusion

A series of 2-coordinate coinage metal (i.e., M = Cu, Ag, Au) complexes bearing a sterically bulky benzimidazolyl-carbene, 1,3-bis(2,6-diisopropylphenyl)-1-H-benzo[d]imidazol-2-ylidene (BZI), and carbazolide (Cz) as the anionic ligand were investigated. X-ray analysis reveals a linear geometry at the metal center with the ligands in a coplanar conformation, as well as orthogonal for **Ag**^BZI^. The redox gap of all the complexes is large (Δ*E*_redox_ > 3 V), in agreement with the high-energy absorption band (> 400 nm in CH_2_Cl_2_) corresponding to the carbazolide-to-carbene ICT transition. The complexes have high luminescence efficiencies (Φ_PL_ >80%) and display deep blue narrow emission in MeCy and PS films. Their absorption spectra display negative solvatochromism, whereas their emission spectra undergo bathochromic shifts in polar solvents that is accompanied by decrease in quantum yields (Φ_PL_ < 23%) and radiative rate constants (*k*_*r*_ < 4.0 × 10^4^ s^−1^). The non-radiative rate constants, however, are unaffected by the medium, remaining nearly the same in polar and nonplar media (*k*_*nr*_ ~ 1 × 10^5^ s^−1^). Temperature-dependent studies reveal that the energy difference between the singlet and triplet excited states in methylcyclohexane is 920 cm^−1^. Vapor-deposited OLEDs fabricated using **Au**^BZI^ as an emissive dopant have high efficiency (EQE = 12%) and a narrow and deep blue emission (CIE = 0.16, 0.06). These two-coordinate complexes present new opportunities for use as dopants in blue OLEDs. Lifetime studies on devices will need to be carried out to determine if these coinage metal-based emitters could serve as alternatives to state-of-the-art Ir(III) complexes commonly used in OLEDs.

## Supporting Information

Synthesis of precursors and complexes, differential pulse voltammetry, cyclic voltammetry curves, photophysical data of the final complexes in various solvents, computational data and x-ray crystallographic data of the final complexes, OLED device fabrication procedure and device characteristics, ^1^H and ^13^C NMR of precursors and final complexes.

## Data Availability Statement

The datasets generated for this study can be found in the Cambridge Crystallographic Data Center (https://www.ccdc.cam.ac.uk/structures/) under the identifiers CuBZI: 1984269, AgBZI: 1984268, and AuBZI: 1984267.

## Author Contributions

RHam synthesized and measured the photophysical properties of the copper, silver, and gold compounds. MI measured and analyzed the temperature dependent photophysical properties of the gold complex. DM carried out the theoretical modeling. MJ prepared and characterized the OLEDs. RHai determined all of the crystal structures. RHam, PD, and MT conceived of the project. MI, PD, and MT wrote the manuscript.

## Conflict of Interest

MT has a financial interest in the Universal Display Corporation. RHam is currently an employee of the Universal Display Corporation, however, all of her work in this paper was carried out when she was a graduate student at the University of Southern California. The remaining authors declare that the research was conducted in the absence of any commercial or financial relationships that could be construed as a potential conflict of interest.
