# Supplementary material for: Highly Efficient Deep Blue Luminescence of 2-Coordinate Coinage Metal Complexes Bearing Bulky NHC Benzimidazolyl Carbene
Source: Front Chem. 2020 May 8;8:401. doi: 10.3389/fchem.2020.00401 (PMC7225363; doi:10.3389/fchem.2020.00401)
Supplement: Supplementary file 1 [file Data_Sheet_1.pdf]

Supporting Information

Highly efficient deep blue luminescence of 2-coordinate coinage metal complexes bearing bulky NHC benzimidazolyl carbene

Rasha Hamze[†‡], Muazzam Idris†, Daniel Sylvinson Muthiah Ravinson, Moon Chul Jung, Ralf Haiges, Peter I. Djurovich, Mark E. Thompson[*]

Department of Chemistry, University of Southern California, Los Angeles, CA, USA

Contents

Synthesis **2**

N^1,N^2-bis(2,6-diisopropylphenyl)benzene-1,2-diamine: 2

1,3-bis(2,6-diisopropylphenyl)-1H-benzo[d]imidazol-3-ium chloride (BzIm.HCl): 3

Chloro[1,3-bis(2,6-diisopropylphenyl)-1-H-benzo[d]imidazol-2-ylidene]copper(I) (**BZICuCl**): 3

Chloro[1,3-bis(2,6-diisopropylphenyl)-1-H-benzo[d]imidazol-2-ylidene]silver(I) (**BZIAgCl**):4

Chloro[1,3-bis(2,6-diisopropylphenyl)-1-H-benzo[d]imidazol-2-ylidene]gold(I) (**BZIAuCl**): 4

Cu^{BZI}: 4

Ag^{BZI}: 5

Au^{BZI}: 5

Electrochemical Measurements: **6**

Photophysical Techniques and Measurements **8**

Molecular Modeling **15**

X-ray crystallographic studies **17**

OLED Fabrication and Characterization **24**

NMR Spectra **28**

References **34**

[†] These authors contributed equally to this paper.

[‡] Current address: Universal Display Corporation, 375 Phillips Blvd, Ewing, NJ 08618

[*] Corresponding author: met@usc.edu

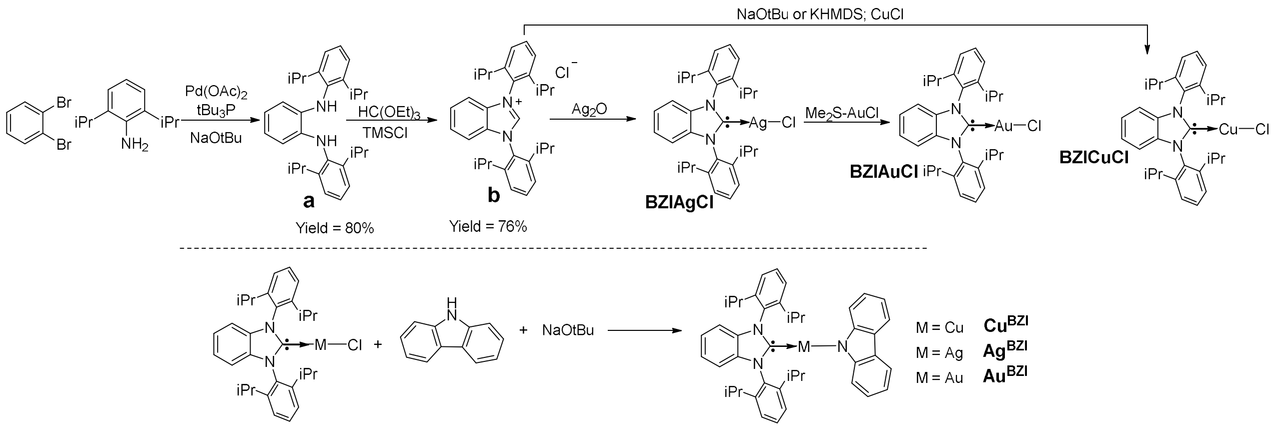

Scheme 1: Synthetic route used in the preparation of two-coordinate coinage metal complexes $\mathbf{M^{BZI}}$ shown below.

Synthesis

All reactions were performed under nitrogen atmosphere in oven dried glassware. Triethyl orthoformate, CuCl, Ag_2O, $(Me_2S)AuCl$, NaOtBu, 9-H-carbazole (Cz), as well as electrochemical grade (tetrabutylammonium fluoride) TBAF, ferrocene (Fc), N,N-Dimethylformamide (DMF), N,N-dibutylformamide (DBF) and decamethyl ferrocene (Fc*) were purchased from Sigma-Aldrich. TMSCl was purchased from TCI Chemicals. All chemicals were used without further purification. Tetrahydrofuran, dichloromethane, and toluene were purified by Glass Contour solvent system by SG Water USA, LLC. Dry, air-free methylcyclohexane (MeCy) and 2-methyltetrahydrofuran (2-MeTHF) were purchased from Sigma-Aldrich and used without further purification. ^{1}H and ^{13}C NMR spectra were recorded on a Varian Mercury 400 and 600. The chemical shifts are given in units of ppm and referenced to the residual proton resonance of chloroform-*d* ($CDCl_3$) at 7.26 ppm. Elemental analyses were performed with Thermo Scientific Flash 2000 at the University of Southern California, CA.

N^1,N^2-bis(2,6-diisopropylphenyl)benzene-1,2-diamine:
In a 500 mL three necked round bottom flask, $Pd(OAc)_2$ (0.305g, 1.36 mmol) and $PtBu_3$ (0.824g, 4.07 mmol) were combined in 80 mL of toluene and stirred under nitrogen until the $Pd(OAc)_2$ dissolved (ca. 5 min.). Dibrobenzene (8.00g, 33.9 mmol) 2,6-diisopropyl aniline (12.0g, 67.8 mmol) and NaOtBu (9.78g, 101.7 mmol) were added to the flask. The reaction mixture was heated to 110 ºC for 16 h. After the reaction, the reaction mixture was quenched with an aqueous concentrated aqueous. The toluene layer was separated and washed 2 x 50 mL with deionized water. The toluene layer was dried over Na_2SO_4, filtered and concentrated under vacuum to yield

a dark oil. Methanol was added to the oil to yield a brown precipitate (11.63g, Yield = 80%). The brown precipitate was filtered and washed with cold methanol, and sublimed (Temperature = 140 °C, Pressure = 1×10^{-6} Torr) to yield white crystals: Overall yield = 8.05 g, 55%. ^{1}H NMR and ^{13}C NMR Spectra match those reported in the literature.[1] The purity of the aniline is crucial in obtaining pure **M^{BZI}** complexes in the last step.

1,3-bis(2,6-diisopropylphenyl)-1H-benzo[d]imidazol-3-ium chloride (BzIm.HCl):
In a 500 mL Schlenk flask equipped with a stir bar and a distillation apparatus, 2.09 g (4.9 mmol) of (N^1,N^2-bis(2,6-diisopropylphenyl)benzene-1,2-diamine was added, and the flask was evacuated then backfilled with N_2 three times. Triethyl orthoformate (300 mL) via cannula, and the orange-colored mixture was stirred for 2h at 150 °C. After ~ 250 mL of triethyl orthoformate was distilled, the temperature was lowered to 50 °C (changed out oil baths), and 60 mL of fresh triethyl orthoformate was added followed by 70 mL of TMSCl. The dark green reaction mixture was stirred at 50 °C for 4 hours, after which the formation of a greyish precipitate was noted. The majority of the organic solvents were removed by vacuum distillation, during which the reaction mixture turned into a wine-red color. The heat was turned off, and the reaction mixture was left to cool under N2. Following that, the precipitate was filtered and washed copiously with diethylether. The filtrate was refiltered, collecting more residue. The light grey-colored product was dried under vacuum. 1.76 g collected (76%). ^{1}H NMR and ^{13}C NMR Spectra match those reported in the literature.[1]

Chloro[1,3-bis(2,6-diisopropylphenyl)-1-H-benzo[d]imidazol-2-ylidene]copper(I) (**BZICuCl**):
In the glovebox, a 100 mL round bottom flask equipped with a stir bar was charged with 430 mg (0.90 mmol) BzIm.HCl and CuCl (90 mg, 0.90 mmol). 30 mL THF was added, followed by NaOtBu (91 mg, 0.95 mmol) which turned the reaction from a white suspension to a pale, golden-colored solution. The reaction mixture was left to stir at room temperature overnight, then filtered through a celite plug. The filtrate was collected, and the volatiles were removed under reduced pressure. The residue was washed copiously with pentane, yielding 422 mg (87% yield) of an off-white powder. ^{1}H NMR (400 MHz, $CDCl_3$) δ 7.57 (t, J = 7.7 Hz, 2H), 7.38 (d, J = 7.6 Hz, 6H), 7.08 (dd, J = 5.7, 3.0 Hz, 2H), 2.40 (hept, J = 13.4, 6.8 Hz, 4H), 1.28 (d, J = 6.8 Hz, 12H), 1.11 (d, J = 6.7 Hz, 12H). ^{13}C NMR (101 MHz, $CDCl_3$) δ 187.39, 146.60, 135.03, 131.73, 131.10, 125.16, 124.77, 111.98, 29.04, 25.16, 23.88. Anal. Calcd for $C_{31}H_{38}ClCuN_2$: C: 69.25; N: 5.21; H: 7.12. Found: C: 66.88; N: 4.90; H:7.09.

Chloro[1,3-bis(2,6-diisopropylphenyl)-1-H-benzo[d]imidazol-2-ylidene]silver(I) (**BZIAgCl**):
In the glovebox, a 200 mL round bottom flask wrapped in aluminum foil and equipped with a stir bar was charged with 850 mg (1.79 mmol) BzIm.HCl and Ag_2O (216 mg, 0.93 mmol). 80 mL THF was added, and the light purplish-colored reaction mixture was left to stir at room temperature overnight, then filtered through a celite plug. The filtrate was collected, and the volatiles were removed under reduced pressure. The residue was washed copiously with pentane, yielding 945 mg of a pale purplish white powder (91% yield). Note: the pale purple tint is likely due to the formation of Ag nanoparticles. To limit this, care must be taken to wrap vials with the solids with aluminum foil and store in the dark under N_2. The two carbene ^{13}C signals observed at 193 ppm and 191 ppm correspond to the two NMR-active Ag isotopes: ^{107}Ag and ^{109}Ag. ^1H NMR (400 MHz, $CDCl_3$) δ 7.58 (t, J = 7.8 Hz, 2H), 7.42 – 7.37 (m, 6H), 7.12 (dd, J = 6.2, 3.1 Hz, 2H), 2.38 (hept, J = 6.8 Hz, 4H), 1.26 (d, J = 6.9 Hz, 12H), 1.10 (d, J = 6.9 Hz, 12H). ^{13}C NMR (101 MHz, $CDCl_3$) δ 193.13, 190.81, 146.59, 135.16, 131.92, 131.29, 125.35, 112.27, 29.01, 25.12, 24.02. Anal. Calcd for $C_{31}H_{38}ClAgN_2$: C: 63.98; N: 4.81; H: 6.58. Found: C: 63.73; N: 4.80; H:6.75.

Chloro[1,3-bis(2,6-diisopropylphenyl)-1-H-benzo[d]imidazol-2-ylidene]gold(I) (**BZIAuCl**):
In the glovebox, a 200 mL round bottom flask wrapped in aluminum foil and equipped with a stir bar was charged with 620 mg (1.07 mmol) **BZIAgCl** and $(Me_2S)AuCl$ (330 mg, 1.12 mmol). 70 mL THF was added, and the light purplish-colored reaction mixture was left to stir at room temperature overnight, then filtered through a celite plug. The filtrate was collected, and the volatiles were removed under reduced pressure. The residue was washed copiously with pentane, yielding 631 mg of a pale purplish white powder (88% yield). Note: the pale purple tint is likely due to the formation of Au nanoparticles. To limit this, care must be taken to wrap vials with the solids with aluminum foil and store in the dark under N_2. ^1H NMR (400 MHz, $CDCl_3$) δ 7.58 (t, J = 7.8 Hz, 2H), 7.40 (dd, J = 6.1, 3.1 Hz, 2H), 7.38 (d, J = 7.8 Hz, 4H), 7.08 (dd, J = 6.1, 3.1 Hz, 2H), 2.40 (hept, J = 6.6 Hz, 4H), 1.33 (d, J = 6.9 Hz, 12H), 1.09 (d, J = 6.9 Hz, 12H). ^{13}C NMR (101 MHz, $CDCl_3$) δ 181.98, 146.64, 134.73, 131.26, 125.54, 124.85, 112.22, 29.14, 24.83, 24.11. Anal. Calcd for $C_{31}H_{38}ClAuN_2$: C: 55.48; N: 4.17; H:5.71. Found: C: 59.99; N: 4.52; H: 6.40.

Cu^{BZI}:
In the glovebox, a 100 mL round bottom flask wrapped in aluminum foil and equipped with a stir bar was charged with 120 mg (0.22 mmol) **BZICuCl**, Cz (39 mg, 0.23 mmol). 30 mL THF was added followed by NaOtBu (22 mg, 0.23 mmol), and the pale orange-colored reaction mixture was

left to stir at room temperature overnight, then filtered through a celite plug. The filtrate was collected, and the volatiles were removed under reduced pressure. The residue was washed copiously with pentane, yielding 75 mg of a white powder (50% yield). ^{1}H NMR (400 MHz, $CDCl_3$) δ 7.88 (d, *J* = 7.8 Hz, 2H), 7.77 – 7.69 (m, 2H), 7.50 (dd, *J* = 7.8, 1.5 Hz, 4H), 7.41 (ddd, *J* = 6.7, 4.9, 2.6 Hz, 2H), 7.22 – 7.16 (m, 2H), 6.99 – 6.90 (m, 2H), 6.84 (t, *J* = 7.3 Hz, 2H), 6.28 (d, *J* = 8.1 Hz, 2H), 2.52 (hept, *J* = 6.9 Hz, 4H), 1.22 (d, *J* = 6.9, 12H), 1.14 (d, *J* = 6.9, 12H). ^{13}C NMR (101 MHz, $CDCl_3$) δ 189.14, 149.88, 147.04, 135.07, 131.97, 130.90, 124.95, 124.74, 123.91, 123.12, 119.26, 115.05, 114.22, 111.76, 29.06, 24.96, 23.86. Anal. Calcd for $C_{43}H_{46}CuN_3$: C: 77.27; N: 6.29; H:6.94. Found: C: 76.11; N: 6.17; H: 6.89.

Ag$^{\mathbf{BZI}}$:
Same as **Cu$^{\mathbf{BZI}}$**. 150 mg (0.26 mmol) **BZIAgCl**, Cz (47 mg, 0.28 mmol), NaOtBu (27 mg, 0.28 mmol), 30 mL THF. 115 mg of a white powder (63% yield). ^{1}H NMR (400 MHz, $CDCl_3$) δ 7.95 (d, *J* = 7.8 Hz, 2H), 7.66 (t, *J* = 7.8 Hz, 2H), 7.48 – 7.42 (m, 6H), 7.21 (q, *J* = 4.1, 3.5 Hz, 2H), 7.05 (ddd, *J* = 8.2, 6.9, 1.3 Hz, 2H), 6.88 (t, *J* = 7.3 Hz, 2H), 6.66 (d, *J* = 8.1 Hz, 2H), 2.51 (hept, *J* = 6.8 Hz, 4H), 1.28 (d, *J* = 6.9 Hz, 12H), 1.14 (d, *J* = 6.9 Hz, 12H). ^{13}C NMR (101 MHz, $CDCl_3$) δ 193.73, 191.70, 150.27, 150.23, 146.71, 139.31, 135.10, 135.03, 132.02, 131.03, 125.74, 125.13, 124.73, 123.89, 123.84, 123.26, 123.10, 120.24, 119.35, 119.33, 114.73, 114.09, 112.06, 110.47, 28.97, 24.86, 23.92. Anal. Calcd for $C_{43}H_{46}AgN_3$: C: 72.46; N: 5.90; H: 6.51. Found: C: 70.83; N: 5.64; H:6.45.

Au$^{\mathbf{BZI}}$:
Same as **Cu$^{\mathbf{BZI}}$**. 784 mg (1.17 mmol) **BZIAuCl**, Cz (195 mg, 1.17 mmol), NaOtBu (118 mg, 1.2 mmol), 80 mL THF. 910 mg of a white powder (97% yield). ^{1}H NMR (400 MHz, $CDCl_3$) δ 7.92 (dt, *J* = 7.8, 1.1 Hz, 2H), 7.70 (t, *J* = 7.8 Hz, 2H), 7.47 (d, *J* = 7.8 Hz, 4H), 7.43 (dd, *J* = 6.1, 3.1 Hz, 2H), 7.20 (dd, *J* = 6.1, 3.1 Hz, 2H), 7.04 (ddd, *J* = 8.2, 7.0, 1.1 Hz, 2H), 6.88 (ddd, *J* = 7.8, 7.0, 1.1 Hz, 2H), 6.71 (dt, *J* = 8.2, 1.1 Hz, 2H), 2.52 (hept, *J* = 6.9 Hz, 4H), 1.31 (d, *J* = 6.9 Hz, 12H), 1.13 (d, *J* = 6.9 Hz, 12H). ^{13}C NMR (101 MHz, $CDCl_3$) δ 185.96, 149.34, 146.93, 134.89, 131.56, 130.91, 125.14, 124.61, 123.67, 123.25, 119.27, 115.63, 113.48, 111.82, 29.09, 24.50, 24.07. Anal. Calcd for $C_{43}H_{46}AuN_3$: C: 64.41; N: 5.24; H: 5.78. Found: C: 63.98; N: 5.16; H:5.81.

Electrochemical Measurements:

Cyclic voltammetry and differential pulsed voltammetry (DPV) were performed using a VersaSTAT 3 potentiostat. Cyclic voltammetry (CV) and differential pulsed voltammetry (DPV) traces are shown on the following page. Anhydrous dimethylformamide (DriSolv) was used as the solvent under inert atmosphere, and 0.1 M tetra(n-butyl)ammonium hexafluorophosphate (TBAF) was used as the supporting electrolyte. A glassy carbon rod was used as the working electrode, a platinum wire was used as the counter electrode, and a silver wire was used as a pseudoreference electrode. Electrochemical reversibility was determined using CV measurements whereas redox potentials are based on values measured using DPV and a ferrocenium/ferrocene (Fc^+/Fc) redox couple used as an internal standard for reductive scans. The oxidation potentials of the $\mathbf{M^{BZI}}$ complexes overlap with that of ferrocene, therefore their oxidation potentials were recorded using decamethylferrocenium/decamethylferrocene ($Me_{10}Fc^+$/$Me_{10}Fc$) as an internal standard and corrected to Fc^+/Fc.

Table S1: Redox potentials of complexes $\mathbf{M^{BZI}}$, and the associated experimental frontier orbital energies.

Complex	E_{ox} (V)	E_{red} (V)	ΔE_{redox} (V)	E_{HOMO} (eV)	E_{LUMO} (eV)
$\mathbf{Cu^{BZI}}$	0.11	-2.85	2.96	-4.92	-1.47
$\mathbf{Ag^{BZI}}$	0.28	-2.84	3.12	-5.12	-1.48
$\mathbf{Au^{BZI}}$	0.29	-2.82	3.14	-5.16	-1.51
CH_2Cl_2	---	-2.73	---	---	---

Electrochemical studies of the $\mathbf{M^{BZI}}$ complexes were performed in dimethylformamide with tetrabutylammonium hexafluorophosphate (TBAF) as the electrolyte, using Fc^+/Fc (reductive scans) and $Me_{10}Fc$ (oxidative scans) as an internal standard. All given values for the potentials are referenced to Fc^+/Fc. The redox peaks were converted to HOMO/LUMO energies using equations in reference 2. The E^*_{red} of $\mathbf{Au^{BZI}}$ is calculated as -2.67 V using $E^*_{red} = E_{red} + \Delta E_{0-0}$.

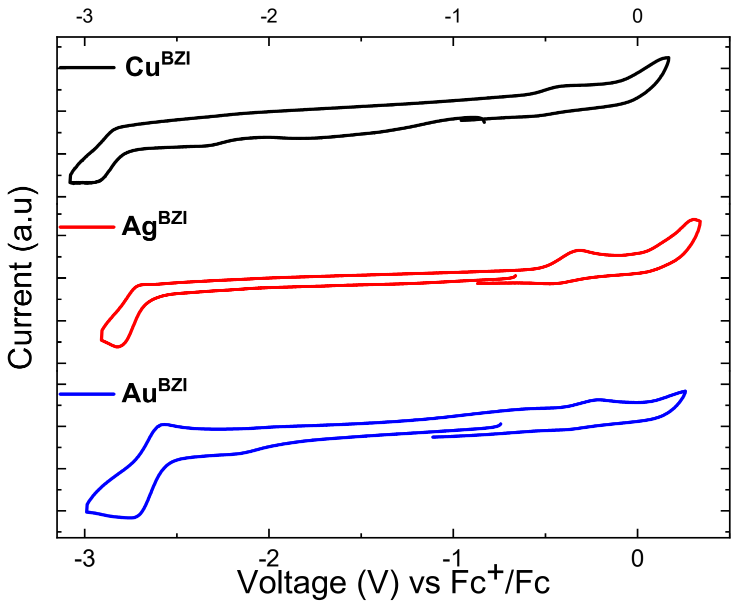

Figure S1: Cyclic voltammetry data of $\mathbf{M^{BZI}}$.

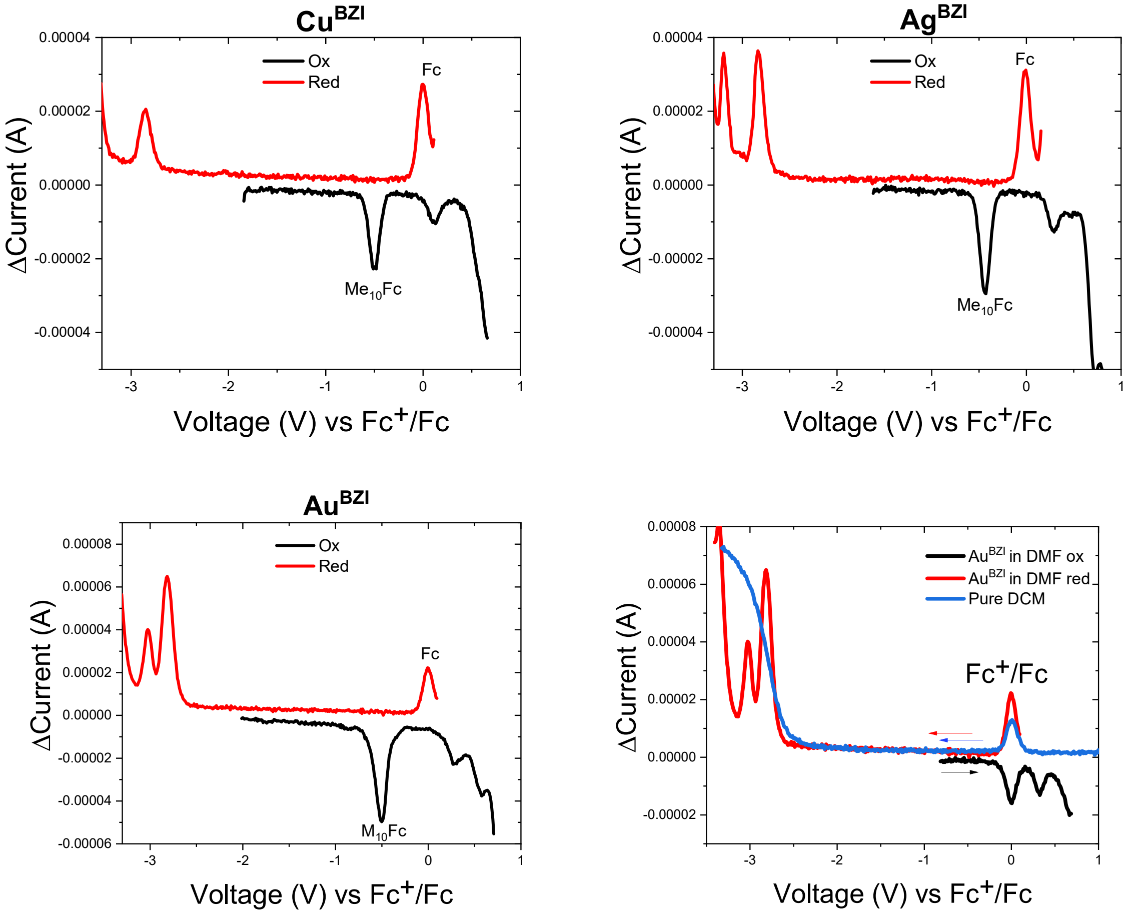

Figure S2: Differential pulse voltammetry data of $\mathbf{M^{BZI}}$ and CH_2Cl_2.

Photophysical Techniques and Measurements

UV–visible spectra were recorded on a Hewlett–Packard 4853 diode array spectrometer. Steady state photoluminescent spectra were measured using a QuantaMaster Photon Technology International phosphorescence/fluorescence spectrofluorometer. Photoluminescent quantum yield (PLQY) measurements were carried out using a Hamamatsu C9920 system equipped with a Xe lamp, calibrated integrating sphere and model C10027 photonic multi-channel analyzer (PMA). All samples in fluid solution were deaerated by extensive sparging with N_2. Temperature-dependent measurements in the range of 80−300 K were performed using an Oxford OptistatDN-V cryostat instrument equipped with an intelligent temperature controller. Doped polystyrene thin films were prepared by first making a solution of polystyrene. Polystyrene pellets (100 mg) were mixed with 2 mL of toluene and sonicated for 1 h until all PS pellets were dissolved. 1 mg of a given metal complex was dissolved in this solution. Of the obtained solution, 0.5 mL was dropcast on a quartz substrate (2 cm x 2 cm) using a pipet to achieve an even surface. The film was left to air-dry for 30 min and then placed in the vacuum chamber for another 30 minutes. The resulting film is approximately 200 microns thick.

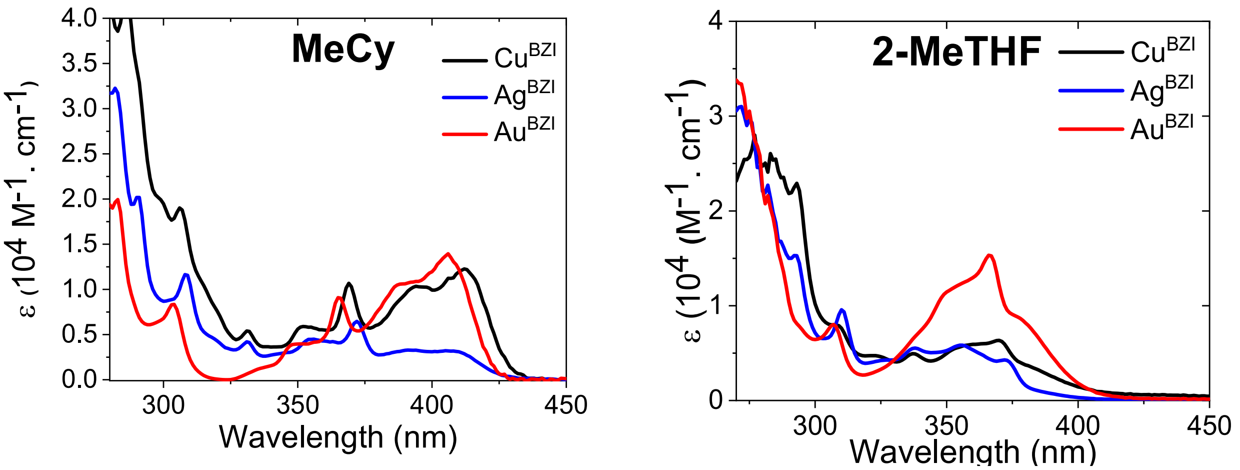

Figure S3: Absorption spectra of $\mathbf{M^{BZI}}$ in MeCy and 2-MeTHF.

Table S2: Absorption wavelengths and molar absorptivities of $\mathbf{M^{BZI}}$ in MeCy and 2-MeTHF at 298 K.

	**MeCy** [nm (ε ×10^4 $M^{-1}\cdot cm^{-1}$)]
$\mathbf{Cu^{BZI}}$	300 (1.88), 330 (0.53), 350 (0.58), 370 (1.05), 395 (1.03), 410 (1.20)
$\mathbf{Ag^{BZI}}$	310 (1.16), 330 (0.42), 355 (0.45), 370 (0.65), 410 (0.30)
$\mathbf{Au^{BZI}}$	305 (0.80), 350 (0.38), 365 (0.90), 390 (1.04), 405 (1.40)
	**2-MeTHF** [nm (ε ×10^4 $M^{-1}\cdot cm^{-1}$)]
$\mathbf{Cu^{BZI}}$	300 (1.00), 340 (0.50), 370 (0.64), 410 (0.08)
$\mathbf{Ag^{BZI}}$	310 (0.97), 340 (0.54), 355 (0.58), 370 (0.40), 410 (0.01)
$\mathbf{Au^{BZI}}$	305 (0.60), 350 (0.81), 365 (1.05), 375 (0.65), 405 (0.13)

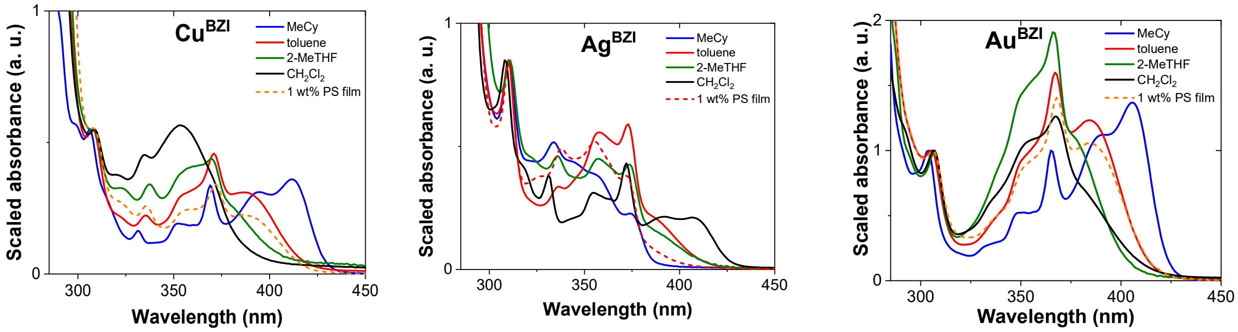

Figure S4: Scaled absorption spectra of complexes $\mathbf{M^{BZI}}$ in various solvents.

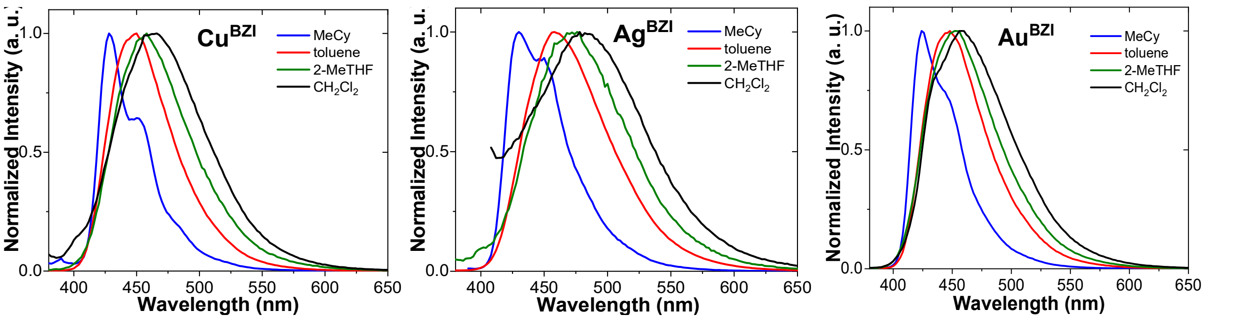

Figure S5: Emission spectra of complexes $\mathbf{M^{BZI}}$ in various solvents.

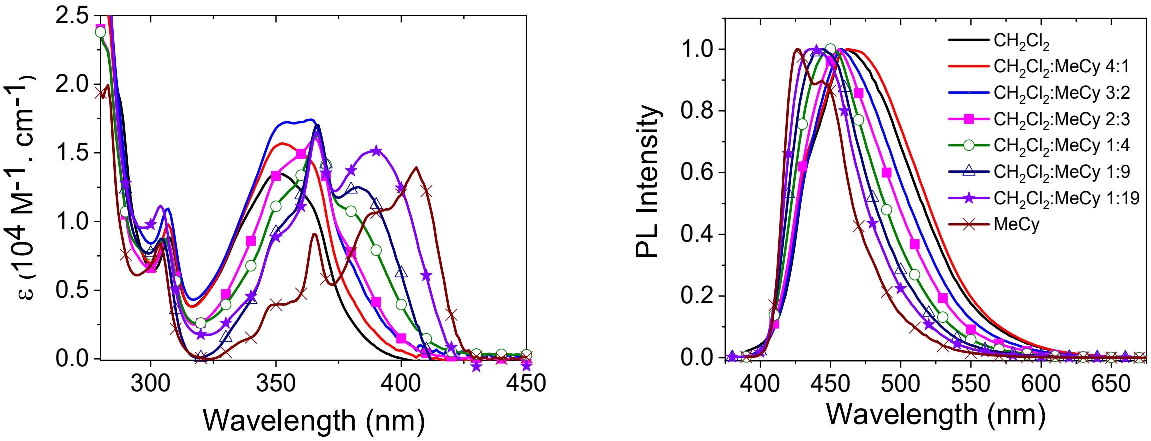

Figure S6: Absorption and emission spectra of complex **Au^{BZI}** in CH_2Cl_2, MeCy and in various solvent ratios.

Table S3: Luminescence properties of **Au^{BZI}** in various CH_2Cl_2:MeCy mixtures.

CH_2Cl_2:MeCy ratio	Φ_{PL}	τ_{RT} (μs)	k_r ($\times 10^5$ s^{-1})	k_{nr} ($\times 10^5$ s^{-1})
1:0	0.23	5.79	0.397	1.33
4:1	0.39	6.17	0.632	0.989
3:2	0.54	5.63	0.959	0.817
2:3	0.69	4.38	1.58	0.708
1:4	0.82	2.47	3.32	0.729
1:9	0.84	1.33	6.32	1.20
1:19	0.87	1.18	7.37	1.10
0:1	0.89	1.15	7.83	0.870

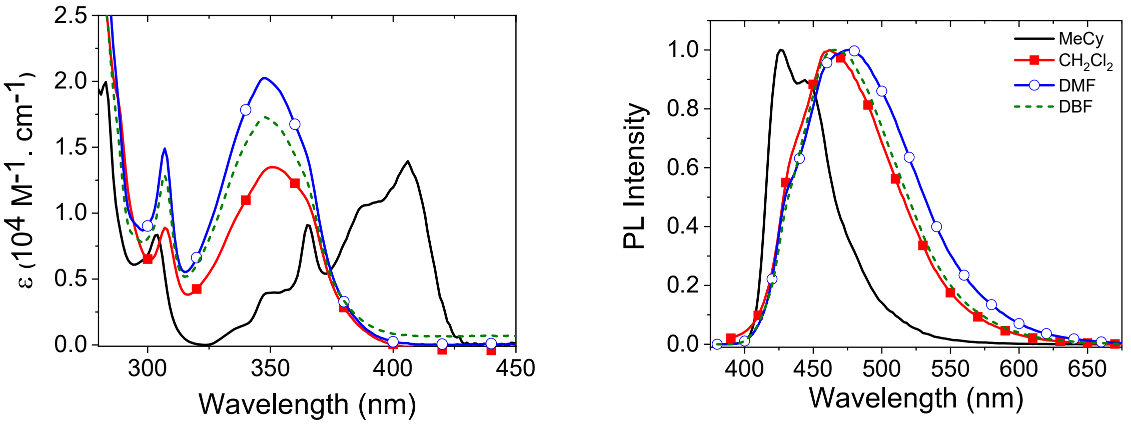

Figure S7: Scaled absorption and emission spectra of complexes **Au^{BZI}** in various solvents (DMF = N,N-Dimethyl formamide, DBF = N,N-Dibutyl formamide).

Table S4: Photophysical properties of **Au^{BZI}** in various solvents.

Solvent	Φ_{PL}	τ_{RT} (μs)	k_r (s^{-1})	k_{nr} (s^{-1})
DBF	0.76	6.24	1.22×10^5	3.85×10^4
DMF	0.30	7.20	4.17×10^4	9.72×10^4
CH_2Cl_2	0.23	5.79	3.97×10^4	1.33×10^5
MeCy	0.89	1.15	7.83×10^5	8.70×10^4

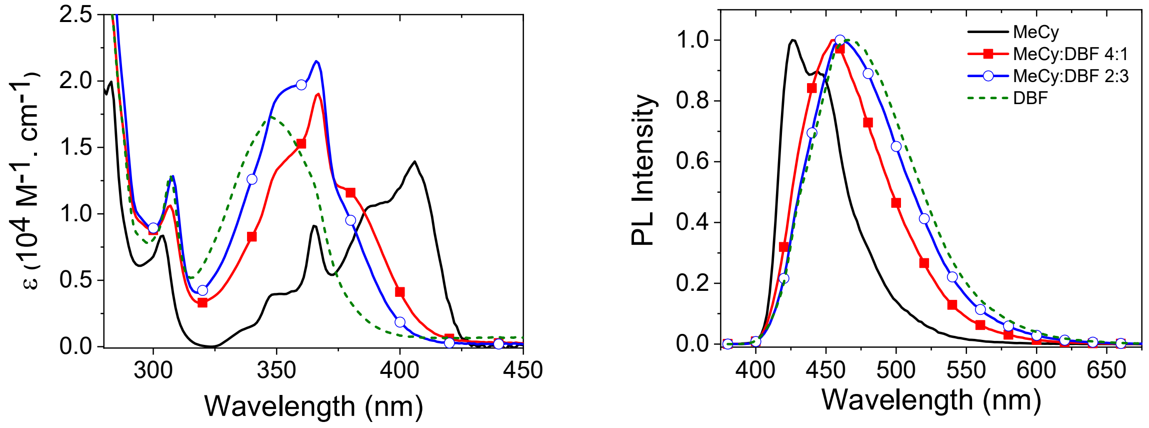

Figure S8: Emission spectra of complex **Au^{BZI}** in DBF, MeCy and in various solvent ratio.

Table S5: Photophysical properties of $\mathbf{Au^{BZI}}$ in DBF, MeCy and in various solvent ratio.

Solvent ratio	Φ_{PL}	τ_{RT} (μs)	k_r (s^{-1})	k_{nr} (s^{-1})
DBF	0.76	6.24	1.22×10^5	3.85×10^4
DBF:MeCy 3:2	0.82	4.30	1.91×10^5	4.19×10^4
DBF:MeCy 1:4	0.88	2.37	3.71×10^5	5.06×10^4
MeCy	0.89	1.15	7.83×10^5	8.70×10^4

The emission spectra of $\mathbf{Au^{BZI}}$ in CH_2Cl_2 decrease in intensity with increasing temperature and show vibronic features at low temperatures (180–230 K) that broaden abruptly at 240 K (Figure S9) The k_r values calculated from the quantum yields measured at various temperatures were fit to a two level model using Equation S1. The fits give an energy difference between the triplet and emitting singlet state of $\mathbf{Au^{BZI}}$ in CH_2Cl_2 as ΔE_{ST} = 730 cm^{-1}, below 250 K. There is also a sharp decrease in k_r at 250 K in CH_2Cl_2, and consequently a smaller energy barrier is observed between 250 K and 270 K (ΔE_{ST} = 175 cm^{-1}). This change in ΔE_{ST} coincides with the marked decrease in efficiency and shift in peak shape observed in the PL spectrum between 240 K and 270 K.

$$ln(k_{TADF}) = ln\left(\frac{k_{ISC}^{S_1\to T_1}}{3}\left(1-\frac{k_{ISC}^{S_1\to T_1}}{k_{fl}+k_{ISC}^{S_1\to T_1}}\right)\right)-\frac{\Delta E_{ST}}{k_B T} \quad \text{(S1)}$$

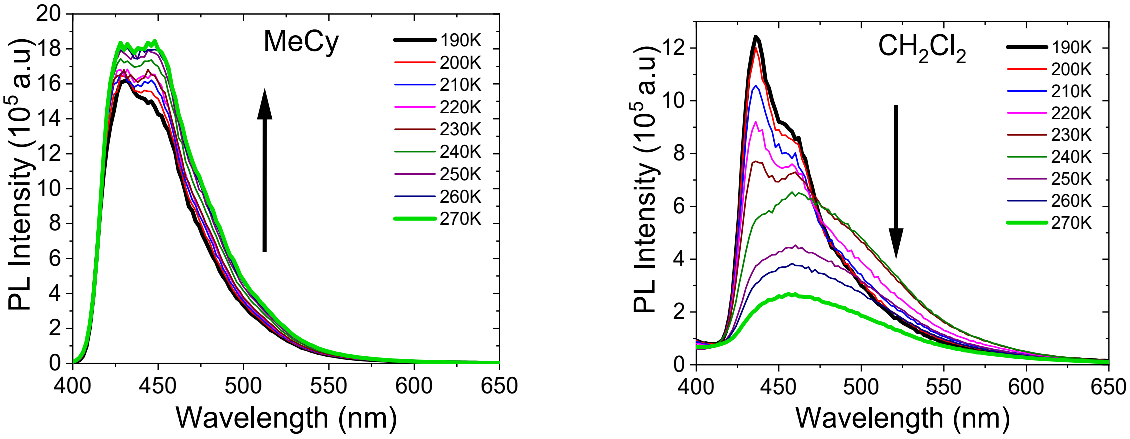

Figure S9: Emission spectra of $\mathbf{Au^{BZI}}$ in CH_2Cl_2 and MeCy at various temperatures.

Table S6: Photophysical properties of **Au^{BZI}** in CH_2Cl_2 at various temperatures

T(°C)	Integrated PL area (×10^7)	Φ_{PL}	τ_{RT} (μs)	k_r (s^{-1})	k_{nr} (s^{-1})
190	7.10	0.59	95.2	6.17×10^3	4.33×10^3
200	7.00	0.58	78.9	7.35×10^3	5.32×10^3
210	6.83	0.57	62.6	9.04×10^3	6.94×10^3
220	6.88	0.57	47.9	1.19×10^4	8.98×10^3
230	7.15	0.59	36.5	1.62×10^4	1.12×10^4
240	6.64	0.55	30.5	1.80×10^4	1.48×10^4
250	4.54	0.38	25.0	1.50×10^4	2.50×10^4
260	3.89	0.32	20.8	1.55×10^4	3.26×10^4
270	2.78	0.23	14.2	1.62×10^4	5.42×10^4

Table S7: Photophysical properties of **Au^{BZI}** in MeCy at various temperatures.

T(°C)	Integrated PL area (×10^8)	Φ_{PL}	τ_{RT} (μs)	k_r (s^{-1})	k_{nr} (s^{-1})
190	1.03	0.71	8.17	8.73×10^4	3.51×10^4
200	1.07	0.74	6.05	1.22×10^5	4.33×10^4
210	1.10	0.76	4.52	1.68×10^5	5.31×10^4
220	1.13	0.78	3.47	2.25×10^5	6.30×10^4
230	1.13	0.78	2.69	2.92×10^5	8.02×10^4
240	1.20	0.83	2.23	3.72×10^5	7.63×10^4
250	1.24	0.86	1.90	4.54×10^5	7.26×10^4
260	1.26	0.88	1.56	5.62×10^5	7.94×10^4
270	1.29	0.89	1.30	6.87×10^5	8.22×10^4

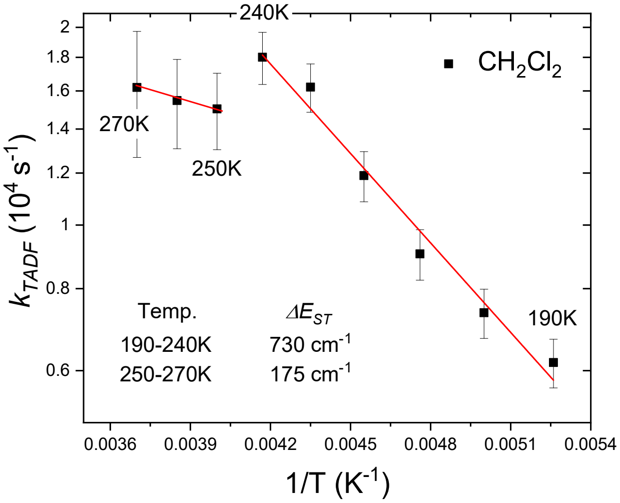

Figure S10: Arrhenius plot of the temperature-dependent lifetime data for **Au**$^{\mathbf{BZI}}$ in CH_2Cl_2 recorded from 180 to 300 K, along with fits to the data according to Equation S1.

Variable temperature ^{1}H NMR of the **Au**$^{\mathbf{BZI}}$ complex in CD_2Cl_2 indicates that the intensity of the residual water peak at temperatures below 240K significantly diminishes and slightly move to downfield suggesting aquo complex formation with **Au**$^{\mathbf{BZI}}$ at temperatures below 240K. This temperature range coincides with the abrupt change observed in emission spectrum and k_r of **Au**$^{\mathbf{BZI}}$ from 230 to 240K. Therefore, these abrupt changes could be due to aquo complex formation of **Au**$^{\mathbf{BZI}}$ with the residual water in CH_2Cl_2.

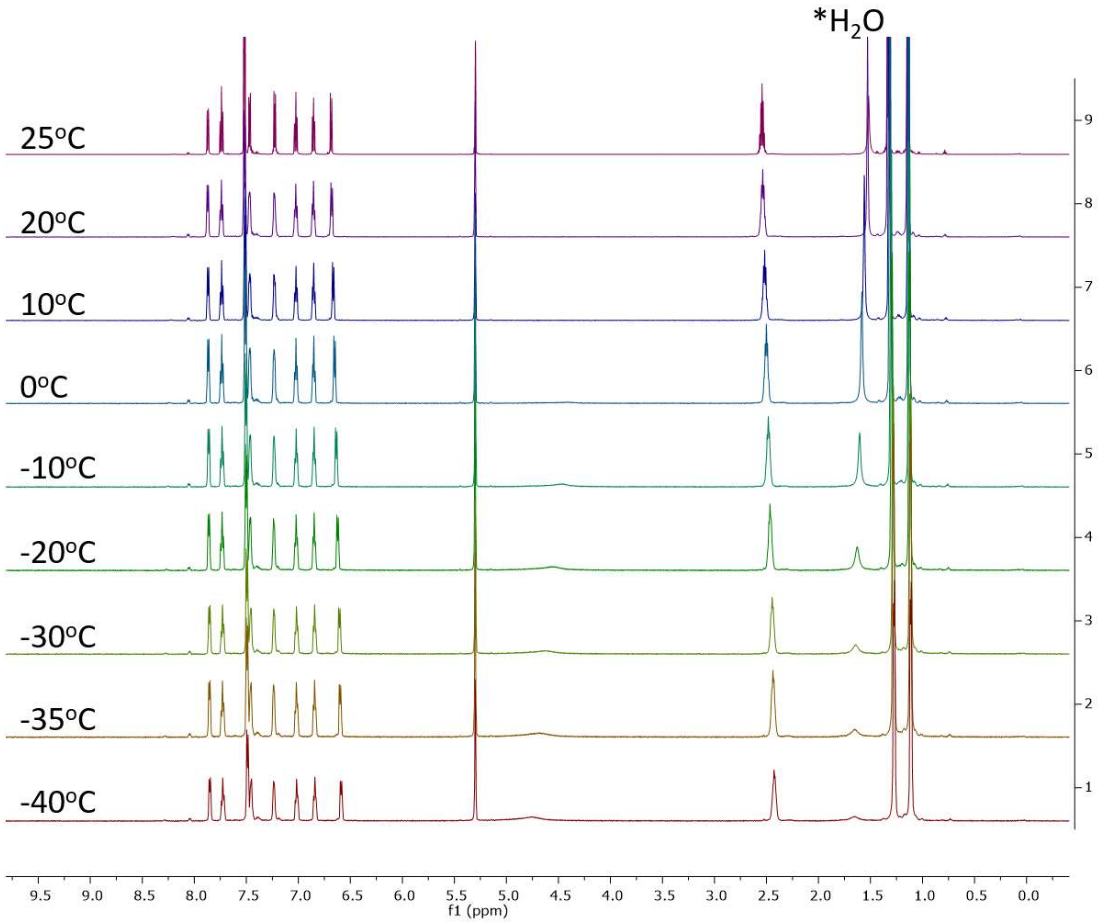

Figure S11: Variable temperature ^{1}H NMR spectra of **Au^{BZI}** in CD_2Cl_2

Molecular Modeling

All Density Functional Theory (DFT) calculations reported in this work were performed using the Q-Chem 5.1 program. Ground state geometry optimization of the complexes were performed at the B3LYP/LACVP** level. Time-dependent density functional theory (TD-DFT) calculations were performed on the ground state optimized geometries at the CAM-B3LYP/LACVP** level for a balanced description of both charge-transfer and locally excited (LE) states.

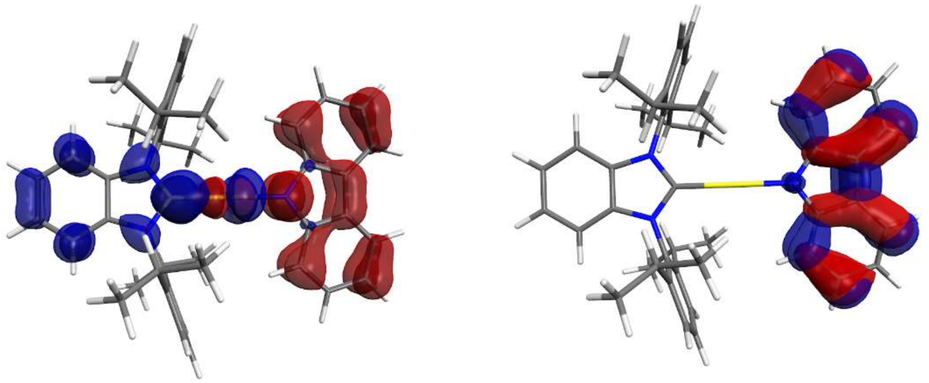

Figure S12: Natural Transition orbitals (NTOs) of ^{1,3}ICT (left) and ^{3}Cz (right) states for the **M^{BZI}** complexes calculated at the CAM-B3LYP/LACVP** level. Hole and electron NTOs are represented by red and blue contours respectively.

Table S8: Calculated singlet and triplet excited state energies and dipole moments for the complexes obtained through TDDFT performed at the CAM-B3LYP/LACVP** level. (Relative contributions of localized Cz and CT character of the transitions are given in parentheses).

	**E (eV)**									f_{S_1}
	**Ground state**	**T_1**	**T_2**	**T_3**	**S_1**	**S_2**	**S_3**	$\Delta E_{^1CT-^3CT}$	$\Delta E_{^3Cz-^3CT}$	
**Cu^{BZI}**		3.05 (Cz)	3.11 (0.6 CT, 0.3 Cz)	3.30 (0.5 CT, 0.5 Cz)	3.37 (CT)	4.12 (0.8 Cz, 0.15 CT)	4.20 (CT)	0.27*	-0.05**	0.132
**μ_{ES} (D)**	-11.9	-11.4	-3.40	-0.6	12.2	-13.4	17.4			
**Ag^{BZIa}**		3.06 (Cz)	3.11 (0.6 Cz, 0.3 CT)	3.36 (0.8 CT, 0.2 Cz)	3.38 (CT)	4.08 (0.8 Cz, 0.15 CT)	4.16 (CT)	0.02*	-0.06**	0.078
**μ_{ES} (D)**	-13.3	-12.8	-10.4	7.8	13.8	-13.6	18.0			
**Ag^{BZIb}**		3.06 (Cz)	3.14 (Cz)	3.32 (CT)	3.33 (CT)	4.04 (Cz)	4.14 (CT)	0.01	0.18	1 x 10^{-6}
**μ_{ES} (D)**	-13.3	-12.9	-15.3	14.3	14.4	-13.6	18.8			
**Au^{BZI}**		3.05 (Cz)	3.14 (0.5 CT, 0.4 Cz)	3.36 (0.6 CT, 0.4 Cz)	3.48 (CT)	4.15 (0.8Cz, 0.15 CT)	4.26 (CT)	0.12*	0.09**	0.200
**μ_{ES} (D)**	-11.8	-11.2	-5.4	1.0	12.1	-13.3	17.1			
	*the energy difference between ^{1}CT (S_1) and the triplet with primarily CT contribution.<br>**the energy difference between ^{3}Cz (T_1) and the closest-lying triplet state with CT contribution.<br>Note: Positive/negative values for the dipole moments indicate that the dipole is directed in/against the Cu-N direction.									

X-ray crystallographic studies

The structures of $\mathbf{M^{BZI}}$ were determined crystallographically. The details of data collection and solution are given below and in cif files provided as supporting materials for this paper.

Table S9: Selected crystallographic data for complexes $\mathbf{M^{BZI}}$.

Complex	C–M (Å)	M–N (Å)	C–N (Å)	dha	C–M–N	Space group	Z
$\mathbf{Cu^{BZI}}$	1.863 (4)	1.869 (3)	3.726 (5)	1.5° (5)	173.9° (2)	Cc	2
$\mathbf{Ag^{BZIa}}$	2.058 (2)	2.050 (2)	4.106 (3)	11.0 (2)	176.15° (7)	P21/n	2
$\mathbf{Ag^{BZIb}}$	2.063 (3)	2.063 (2)	4.125 (4)	95.0° (2)	177.66° (8)		
$\mathbf{Au^{BZI}}$	1.981 (4)	2.017 (4)	3.997 (6)	3.2 (5)	176.4 (2)	C2/c	1

[a] C–M and M–N refer to the carbene–metal and metal–carbazole bond lengths respectively. dha refers to the carbene-carbazole dihedral angle.

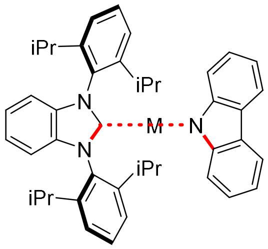

Illustration of carbene-carbazole dihedral angle

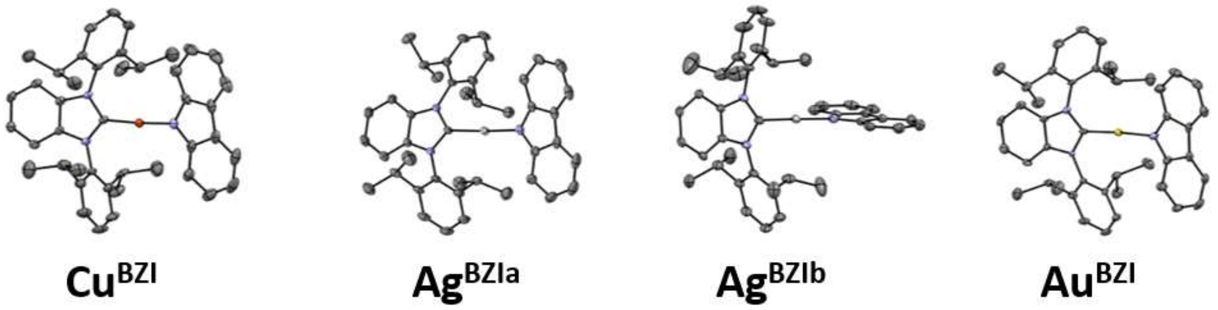

Figure S13: Thermal ellipsoid diagrams representing the crystal structures of $\mathbf{M^{BZI}}$ and conformers of complex Ag^{BZI}.

Structural analysis of $\mathbf{Cu^{BZI}}$

A clear yellow rod-like specimen of $C_{43}H_{46}CuN_3$, approximate dimensions 0.050 mm x 0.050 mm x 0.210 mm, was used for the X-ray crystallographic analysis. The X-ray intensity data were

measured on a Bruker APEX DUO system equipped with a fine-focus tube (MoKα , λ = 0.71073 Å) and a TRIUMPH curved-crystal monochromator.

The total exposure time was 10.40 hours. The frames were integrated with the Bruker SAINT software package using a SAINT V8.38A (Bruker AXS, 2013) algorithm. The integration of the data using a monoclinic unit cell yielded a total of 46026 reflections to a maximum θ angle of 30.45° (0.70 Å resolution), of which 17730 were independent (average redundancy 2.596, completeness = 92.8%, R_{int} = 4.49%, R_{sig} = 6.32%) and 14703 (82.93%) were greater than 2σ(F^2). The final cell constants of a = 21.514(8) Å, b = 19.882(7) Å, c = 17.357(6) Å, β = 98.281(6)°, volume = 7347.(5) $Å^3$, are based upon the refinement of the XYZ-centroids of 9944 reflections above 20 σ(I) with 4.734° < 2θ < 60.17°. Data were corrected for absorption effects using the multi-scan method (SADABS). The ratio of minimum to maximum apparent transmission was 0.850. The calculated minimum and maximum transmission coefficients (based on crystal size) are 0.8800 and 0.9690.

The structure was solved and refined using the Bruker SHELXTL Software Package, using the space group C 1 c 1, with Z = 8 for the formula unit, $C_{43}H_{46}CuN_3$. The final anisotropic full-matrix least-squares refinement on F^2 with 864 variables converged at R1 = 4.45%, for the observed data and wR2 = 10.49% for all data. The goodness-of-fit was 1.027. The largest peak in the final difference electron density synthesis was 1.028 $e^-/Å^3$ and the largest hole was -0.546 $e^-/Å^3$ with an RMS deviation of 0.065 $e^-/Å^3$. On the basis of the final model, the calculated density was 1.208 g/cm^3 and F(000), 2832 e^-.

Sample and crystal data for Cu^{BZI}

**Identification code**	Cu^{BZI} _Et2O	
**Chemical formula**	$C_{43}H_{46}CuN_3$	
**Formula weight**	668.37 g/mol	
**Temperature**	100(2) K	
**Wavelength**	0.71073 Å	
**Crystal size**	0.050 x 0.050 x 0.210 mm	
**Crystal habit**	clear yellow rod	
**Crystal system**	monoclinic	
**Space group**	C 1 c 1	
**Unit cell dimensions**	a = 21.514(8) Å	α = 90°
	b = 19.882(7) Å	β = 98.281(6)°
	c = 17.357(6) Å	γ = 90°
**Volume**	7347.(5) $Å^3$	
**Z**	8	
**Density (calculated)**	1.208 g/cm^3	
**Absorption coefficient**	0.627 mm^{-1}	
**F(000)**	2832	

Data collection and structure refinement for Cu^{BZI}_Et2O

**Diffractometer**	Bruker APEX DUO	
**Radiation source**	fine-focus tube (MoKα , λ = 0.71073 Å)	
**Theta range for data collection**	1.74 to 30.45°	
**Index ranges**	-29<=h<=30, -27<=k<=28, -24<=l<=24	
**Reflections collected**	46026	
**Independent reflections**	17730 [R(int) = 0.0449]	
**Coverage of independent reflections**	92.8%	
**Absorption correction**	multi-scan	
**Max. and min. transmission**	0.9690 and 0.8800	
**Structure solution technique**	direct methods	
**Structure solution program**	SHELXTL XT 2014/5 (Bruker AXS, 2014)	
**Refinement method**	Full-matrix least-squares on F^2	
**Refinement program**	SHELXTL XL 2018/3 (Bruker AXS, 2018)	
**Function minimized**	Σ w(F_o^2 - F_c^2)^2	
**Data / restraints / parameters**	17730 / 2 / 864	
**Goodness-of-fit on F^2**	1.027	
**Δ/σ_{max}**	0.001	
**Final R indices**	14703 data; $I>2\sigma(I)$	R1 = 0.0445, wR2 = 0.0985
	all data	R1 = 0.0609, wR2 = 0.1049
**Weighting scheme**	$w=1/[\sigma^2(F_o^2)+(0.0520P)^2+1.2758P]$ where $P=(F_o^2+2F_c^2)/3$	
**Absolute structure parameter**	0.313(10)	
**Largest diff. peak and hole**	1.028 and -0.546 eÅ^{-3}	
**R.M.S. deviation from mean**	0.065 eÅ^{-3}	

Structural analysis of Ag^{BZI}

A clear colourless prism-like specimen of $C_{43}H_{46}AgN_3$, approximate dimensions 0.158 mm x 0.410 mm x 0.495 mm, was used for the X-ray crystallographic analysis. The X-ray intensity data were measured on a Bruker APEX DUO system equipped with a TRIUMPH curved-crystal monochromator and a MoKα fine-focus tube (λ = 0.71073 Å).

The total exposure time was 1.40 hours. The frames were integrated with the Bruker SAINT software package using a SAINT V8.38A (Bruker AXS, 2013) algorithm. The integration of the data using a monoclinic unit cell yielded a total of 183521 reflections to a maximum θ angle of 30.57° (0.70 Å resolution), of which 23671 were independent (average redundancy 7.753, completeness = 99.0%, R_{int} = 9.47%, R_{sig} = 5.97%) and 18836 (79.57%) were greater than $2\sigma(F^2)$. The final cell constants of a = 22.904(15) Å, b = 16.422(11) Å, c = 23.154(15) Å, β = 116.620(10)°, volume = 7786.(9) $Å^3$, are based upon the refinement of the XYZ-centroids of 9171 reflections above 20 σ(I) with 4.349° < 2θ < 60.35°. Data were corrected for absorption effects using the multi-scan method (SADABS). The ratio of minimum to maximum apparent transmission was 0.835. The calculated minimum and maximum transmission coefficients (based on crystal size) are 0.7730 and 0.9180. The structure was solved and refined using the Bruker SHELXTL Software Package, using the space group P 1 21/n 1, with Z = 8 for the formula unit, $C_{43}H_{46}AgN_3$. The final anisotropic full-matrix least-squares refinement on F^2 with 863 variables converged at R1 = 3.71%, for the observed data and wR2 = 9.94% for all data. The goodness-of-fit was 1.015. The largest peak in the final difference electron density synthesis was 1.000 $e^-/Å^3$ and the largest hole was -0.688 $e^-/Å^3$ with an RMS deviation of 0.081 $e^-/Å^3$. On the basis of the final model, the calculated density was 1.216 g/cm^3 and F(000), 2976 e^-.

Sample and crystal data for Ag^{BZI}

**Identification code**	Ag^{BZI}	
**Chemical formula**	$C_{43}H_{46}AgN_3$	
**Formula weight**	712.70 g/mol	
**Temperature**	100(2) K	
**Wavelength**	0.71073 Å	
**Crystal size**	0.158 x 0.410 x 0.495 mm	
**Crystal habit**	clear colourless prism	
**Crystal system**	monoclinic	
**Space group**	P 1 21/n 1	
**Unit cell dimensions**	a = 22.904(15) Å	α = 90°
	b = 16.422(11) Å	β = 116.620(10)°
	c = 23.154(15) Å	γ = 90°
**Volume**	7786.(9) $Å^3$	
**Z**	8	
**Density (calculated)**	1.216 g/cm^3	

**Absorption coefficient**	0.549 mm^{-1}
**F(000)**	2976

Data collection and structure refinement for Ag^{BZI}

**Diffractometer**	Bruker APEX DUO	
**Radiation source**	fine-focus tube, MoKα	
**Theta range for data collection**	1.04 to 30.57°	
**Index ranges**	-32<=h<=32, -23<=k<=23, -32<=l<=33	
**Reflections collected**	183521	
**Independent reflections**	23671 [R(int) = 0.0947]	
**Coverage of independent reflections**	99.0%	
**Absorption correction**	multi-scan	
**Max. and min. transmission**	0.9180 and 0.7730	
**Structure solution technique**	direct methods	
**Structure solution program**	SHELXTL XT 2014/5 (Bruker AXS, 2014)	
**Refinement method**	Full-matrix least-squares on F^2	
**Refinement program**	SHELXTL XL 2016/6 (Bruker AXS, 2016)	
**Function minimized**	$\Sigma w(F_o^2 - F_c^2)^2$	
**Data / restraints / parameters**	23671 / 0 / 863	
**Goodness-of-fit on F^2**	1.015	
**Δ/σ_{max}**	0.003	
**Final R indices**	18836 data; I>2σ(I)	R1 = 0.0371, wR2 = 0.0899
	all data	R1 = 0.0527, wR2 = 0.0994
**Weighting scheme**	$w=1/[\sigma^2(F_o^2)+(0.0312P)^2+4.9176P]$ where $P=(F_o^2+2F_c^2)/3$	
**Largest diff. peak and hole**	1.000 and -0.688 $eÅ^{-3}$	
**R.M.S. deviation from mean**	0.081 $eÅ^{-3}$	

Structural analysis of Au^{BZI}

A clear colourless plate-like specimen of $C_{44}H_{48}AuCl_2N_3$, approximate dimensions 0.080 mm x 0.250 mm x 0.340 mm, was used for the X-ray crystallographic analysis. The X-ray intensity data were measured on a Bruker APEX DUO system equipped with a fine-focus tube (MoKα , λ = 0.71073 Å) and a TRIUMPH curved-crystal monochromator.

The total exposure time was 1.40 hours. The frames were integrated with the Bruker SAINT software package using a SAINT V8.38A (Bruker AXS, 2013) algorithm. The integration of the data using a monoclinic unit cell yielded a total of 78978 reflections to a maximum θ angle of 27.48° (0.77 Å resolution), of which 9166 were independent (average redundancy 8.616, completeness = 100.0%, R_{int} = 6.32%, R_{sig} = 3.34%) and 8254 (90.05%) were greater than $2\sigma(F^2)$. The final cell constants of a = 24.594(7) Å, b = 8.764(2) Å, c = 37.333(10) Å, β = 97.418(4)°, volume = 7979.(4) $Å^3$, are based upon the refinement of the XYZ-centroids of 9358 reflections above 20 σ(I) with 4.401° < 2θ < 56.68°. Data were corrected for absorption effects using the multi-scan method (SADABS). The ratio of minimum to maximum apparent transmission was 0.567. The calculated minimum and maximum transmission coefficients (based on crystal size) are 0.0321 and 0.0656.

The structure was solved and refined using the Bruker SHELXTL Software Package, using the space group C 1 2/c 1, with Z = 8 for the formula unit, $C_{44}H_{48}AuCl_2N_3$. The final anisotropic full-matrix least-squares refinement on F^2 with 459 variables converged at R1 = 3.64%, for the observed data and wR2 = 6.81% for all data. The goodness-of-fit was 1.230. The largest peak in the final difference electron density synthesis was 1.781 $e^-/Å^3$ and the largest hole was -2.769 $e^-/Å^3$ with an RMS deviation of 0.102 $e^-/Å^3$. On the basis of the final model, the calculated density was 1.476 g/cm^3 and F(000), 3568 e^-.

Sample and crystal data for Au^{BZI} _CH_2Cl_2

**Identification code**	Au^{BZI} _CH_2Cl_2	
**Chemical formula**	$C_{44}H_{48}AuCl_2N_3$	
**Formula weight**	886.72 g/mol	
**Temperature**	100(2) K	
**Wavelength**	0.71073 Å	
**Crystal size**	0.080 x 0.250 x 0.340 mm	
**Crystal habit**	clear colourless plate	
**Crystal system**	monoclinic	
**Space group**	C 1 2/c 1	
**Unit cell dimensions**	a = 24.594(7) Å	α = 90°
	b = 8.764(2) Å	β = 97.418(4)°
	c = 37.333(10) Å	γ = 90°
**Volume**	7979.(4) $Å^3$	
**Z**	8	

**Density (calculated)**	1.476 g/cm^3
**Absorption coefficient**	3.855 mm^{-1}
**F(000)**	3568

Data collection and structure refinement for Au^{BZI} CH_2Cl_2

**Diffractometer**	Bruker APEX DUO	
**Radiation source**	fine-focus tube (MoKα , λ = 0.71073 Å)	
**Theta range for data collection**	1.10 to 27.48°	
**Index ranges**	-31<=h<=31, -11<=k<=11, -48<=l<=48	
**Reflections collected**	78978	
**Independent reflections**	9166 [R(int) = 0.0632]	
**Coverage of independent reflections**	100.0%	
**Absorption correction**	multi-scan	
**Max. and min. transmission**	0.0656 and 0.0321	
**Structure solution technique**	direct methods	
**Structure solution program**	SHELXTL XT 2014/5 (Bruker AXS, 2014)	
**Refinement method**	Full-matrix least-squares on F^2	
**Refinement program**	SHELXTL XL 2018/3 (Bruker AXS, 2018)	
**Function minimized**	Σ w(F_o^2 - F_c^2)^2	
**Data / restraints / parameters**	9166 / 0 / 459	
**Goodness-of-fit on F^2**	1.230	
**Δ/σ_{max}**	0.002	
**Final R indices**	8254 data; I>2σ(I)	R1 = 0.0364, wR2 = 0.0664
	all data	R1 = 0.0429, wR2 = 0.0681
**Weighting scheme**	w=1/[σ^2(F_o^2)+36.5935P] where P=(F_o^2+2F_c^2)/3	
**Largest diff. peak and hole**	1.781 and -2.769 eÅ^{-3}	
**R.M.S. deviation from mean**	0.102 eÅ^{-3}	

OLED Fabrication and Characterization

OLED devices were fabricated on pre-patterned ITO-coated glass substrates (20 ± 5 Ω cm2, Thin Film Devices, Inc.). Prior to deposition, the substrates were cleaned with soap, rinsed with deionized water and sonicated for 15 minutes. Afterwards, two subsequent rinses and 12-minute sonication baths were performed in acetone and isopropyl alcohol sequentially, followed by 10 min UV ozone exposure. All organic layers as well as the Al cathode were deposited in a vacuum thermal evaporator, EVO Vac 800 deposition system from Angstrom Engineering, at 6 x 10^{-7} Torr. Current-voltage-luminescence (J-V-L) curves were derived by using a Keithley power source meter model 2400 and a Newport multifunction optical model 1835-C, PIN-220DP/SB blue enhanced silicon photodiodes (OSI optoelectronics Ltd.). The sensor was set to measure power at an energy of 520 nm, followed by correcting to the average electroluminescence wavelength for each individual device during data process. Electroluminescence (EL) spectra of OLEDs were measured using the fluorimeter (model C-60 Photon Technology International QuantaMaster) at several different voltages.

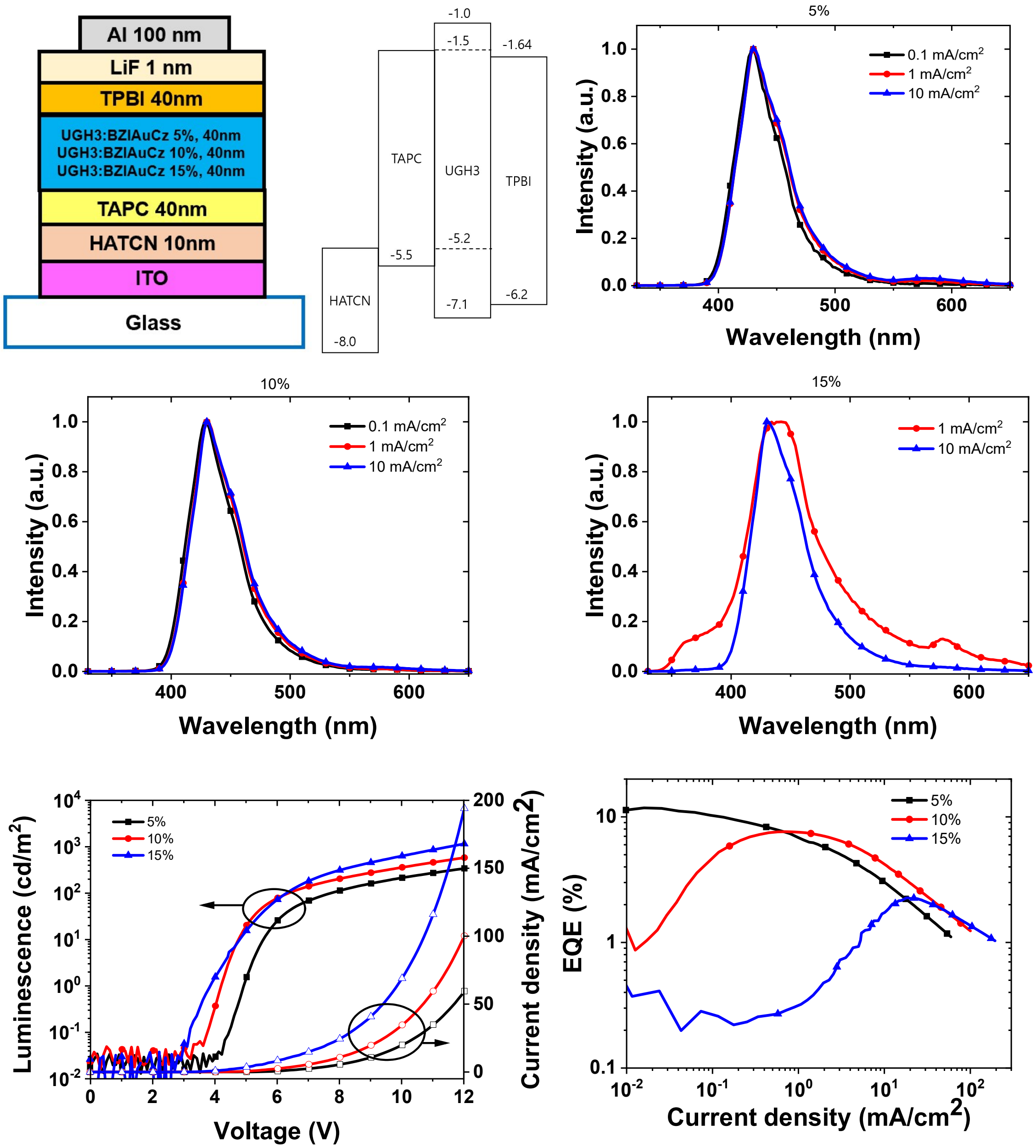

Figure S14: Electroluminescent device characteristics **Au^{BZI}** with doping concentrations of 5%, 10% and 15% (a) energies of frontier orbital in eV. (b) Electroluminescent spectra of the devices. (c) Current density- Voltage-Luminescence (J-V-L) curve. (d) External Quantum Efficiency (EQE) curve. Inset: Molecular structures of materials utilized in the devices.

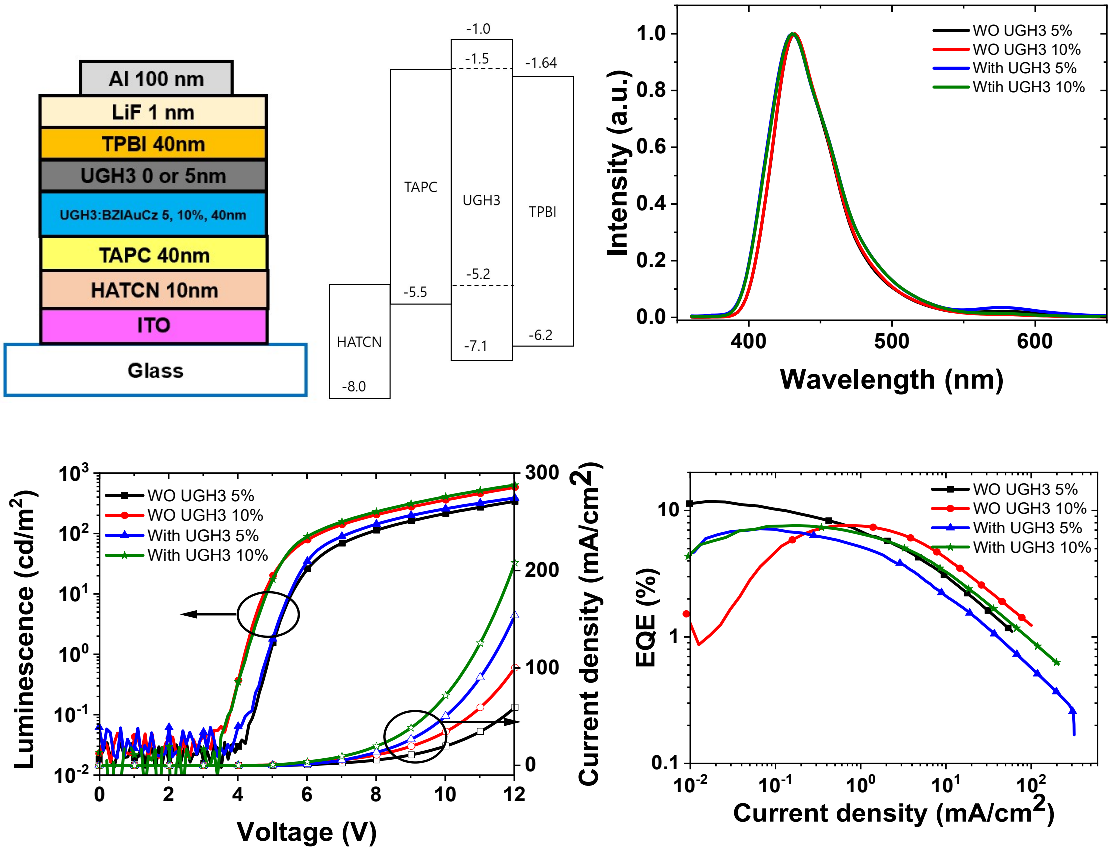

Figure S15: devices with 5 and 10% doping without (WO) and with UGH3 blocker (4 devices)

Considering the high triplet energy of **Au**^{BZI} (E_T = 3.1 eV), 1,3-bis(triphenylsilyl)benzene (UGH3) is employed in the device as a host due to its higher triplet energy (E_T = 3.5 eV). 1,4,5,8,9,11-hexaazatriphenylene hexacarbonitrile (HATCN), 4,4′cyclohexylidenebis[N,N-bis(4-methylphenyl)benzenamine (TAPC) and 2,2′,2″-(1,3,5-benzinetriyl)-tris(1-phenyl-1-H-benzimidazole] (TPBI) were utilized as hole injection (HIL), hole transporting (HTL) and electron transporting Layer (ETL) respectively. As shown in Figure S9, the devices with **Au**^{BZI} dopedat different levels (5,10 and 15 %) were fabricated to investigate the effect of doping concentration on device performances. The EL (Figure S9) spectra at different doping ratios and current densities show that the stability of the devices is dependent on both doping conditions and current densities. Unlike the device with 10 % doping ratio where the EL spectra are relatively stable, devices doped at 5 % and 15 % have slight undesirable emission at 550 nm. The emission at 550 nm becomes more intense after the second and third scans of the devices, suggesting that this emission is likely

due to device degradation. Devices doped at 10% however exhibit consistent emission profile at different current density with no emission at 550 nm, suggesting that degradation of the device at 10% doping level is significantly prohibited. Current density-Voltage-Luminance (J-V-L) curve shows increase in conductivity and decrease in turn-on voltage of the devices as doping concentration increases. This is consistent with the fact that the HOMO/LUMOs of the emitter are entirely nested by that of UGH3, allowing the charges to be injected and carried by the dopant. Therefore, increasing doping concentration increases more pathways for charges to be carried and hence increase in conductivity. The efficiencies of these devices are moderate with maximum EQE of 10 % and 5 % doped devices of 7.7 % and 11.8 % respectively. To improve the efficiency of the 5% and 10% doped devices, thin layer (5 nm) of wide bandgap UGH3 is introduced as exciton/hole blocking layer between the EML and ETL. This ensures exciton and hole confinement in the EML, thereby preventing exciton quenching by TPBi. Figure S10 shows the architecture and performances of four different devices: 5 % and 10 % doping condition was selected with the absence or presence of UGH3 as an exciton blocker. Similar to doping concentration experiments, the EL spectra of the 5 % doping exhibits a peak at 550 nm, but the peak is absent in the 10% devices. The turn-on voltage of the devices stays the same, whereas the conductivity of the devices increases when UGH3 blocker is introduced. These observations are counter intuitive considering higher LUMO level of UGH3 compared to that of the dopant. Currently, we do not have an explicit experimental data to interpret the result, but one plausible explanation might be related to large permanent dipole moment of **Au$^{\mathbf{BZI}}$**. It is well reported that several molecules with high permanent dipole moment happen to form polarized charges at the interface, influencing on the device properties such as charge injection and transporting ability[3].[4, 5]Large dipole moment of **Au$^{\mathbf{BZI}}$** forms at the EML/HTL, EML/ETL interfaces such a way that is favorable for charges to be injected to EML, leading to unchanged turn on voltage even when UGH3 is introduced. Surprisingly, the EQE did not improve with the introduction of UGH3, implying that exciton quenching or hole leakage through TPBi does not occur.

NMR Spectra

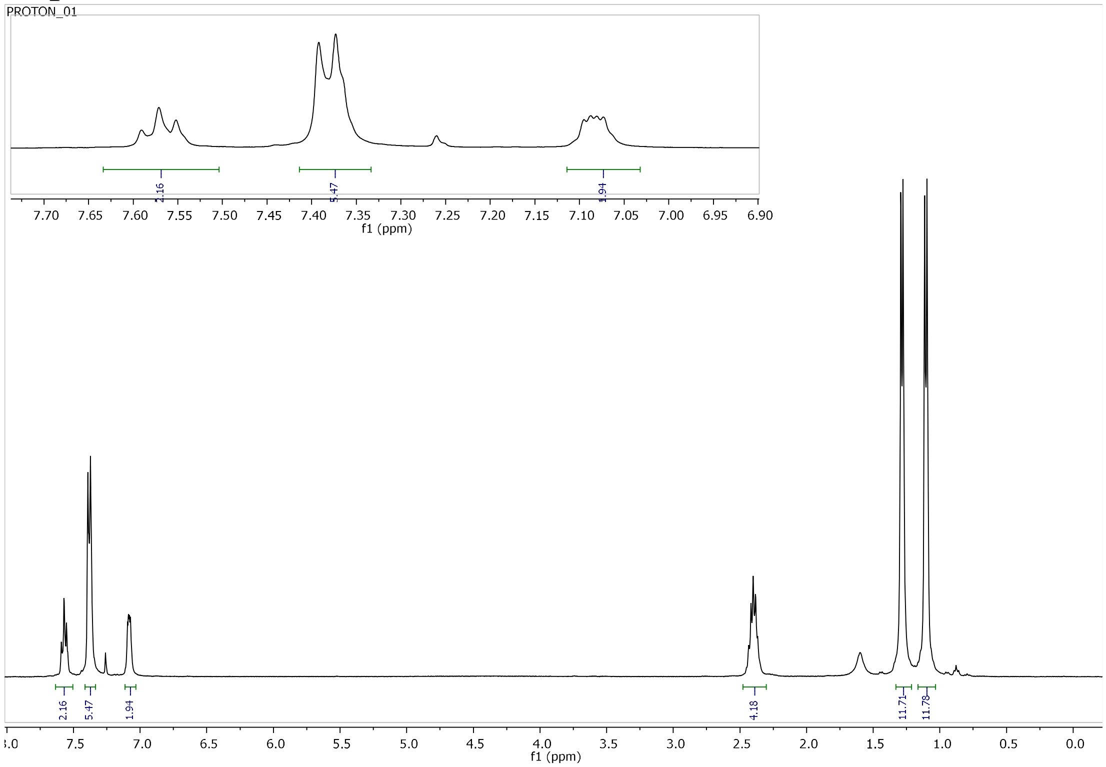

Figure S16: ^{1}H NMR spectrum of **(BZI)CuCl** in $CDCl_3$

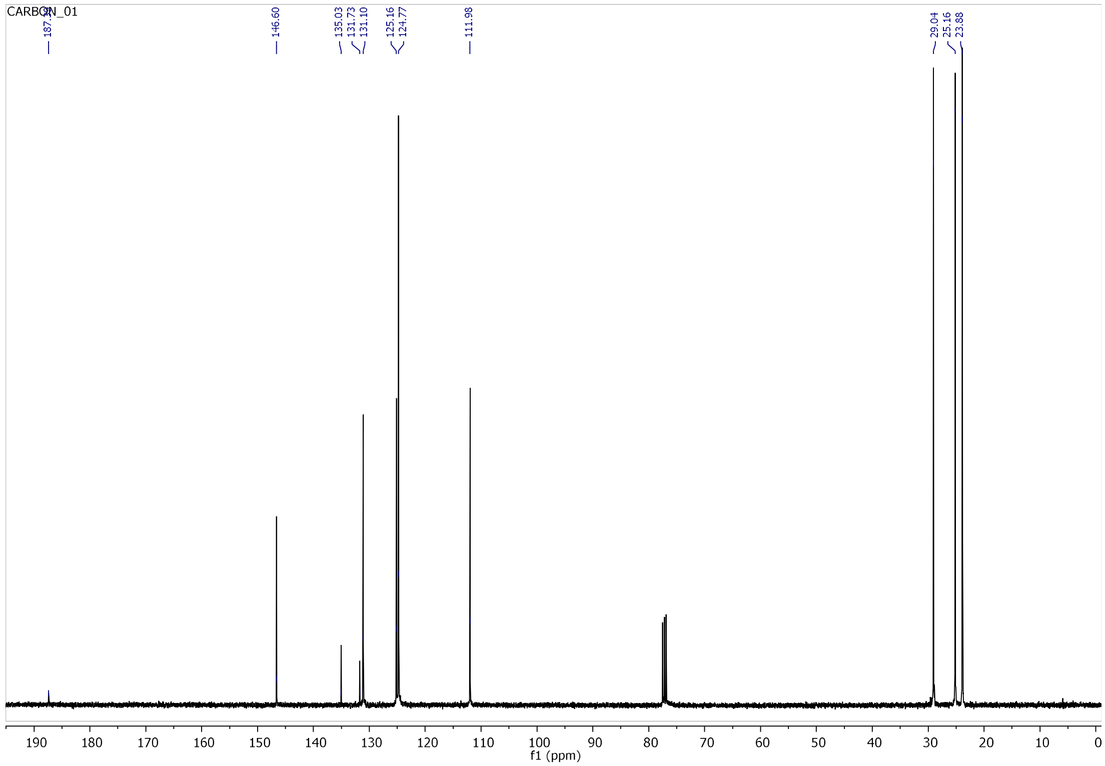

Figure S17: ^{13}C NMR spectrum of **b(BZI)CuCl** in $CDCl_3$

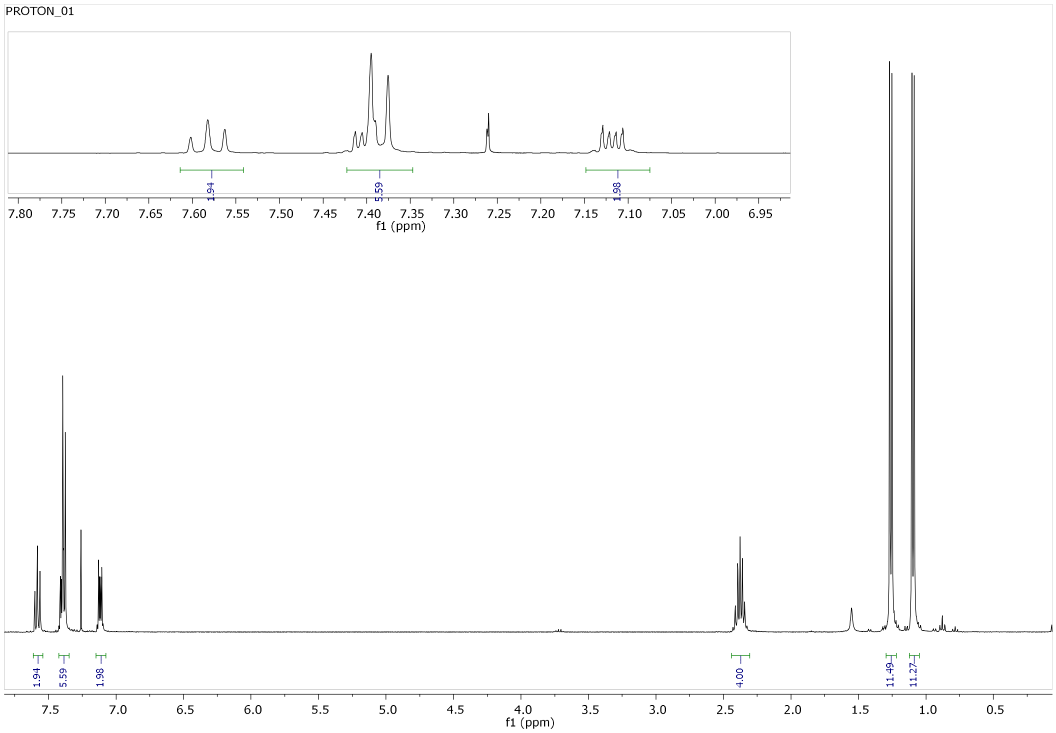

Figure S18: ^{1}H NMR spectrum of **(BZI)AgCl** in $CDCl_3$

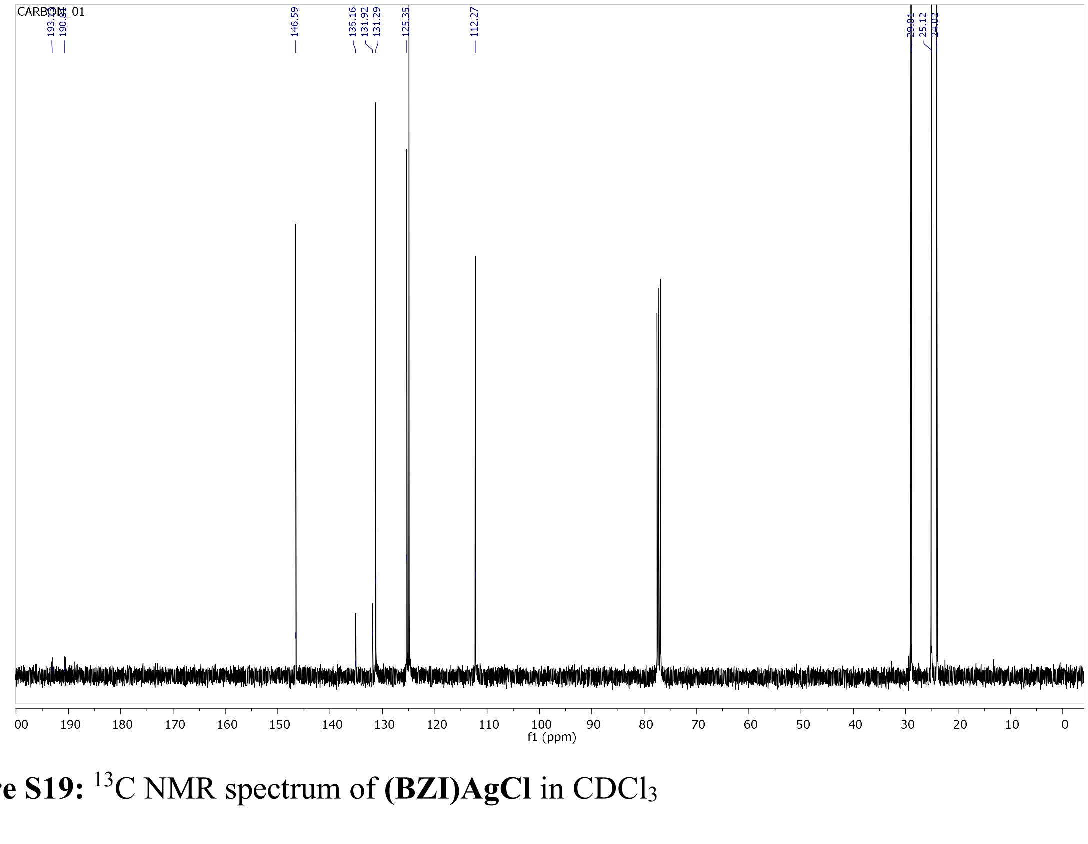

Figure S19: ^{13}C NMR spectrum of **(BZI)AgCl** in $CDCl_3$

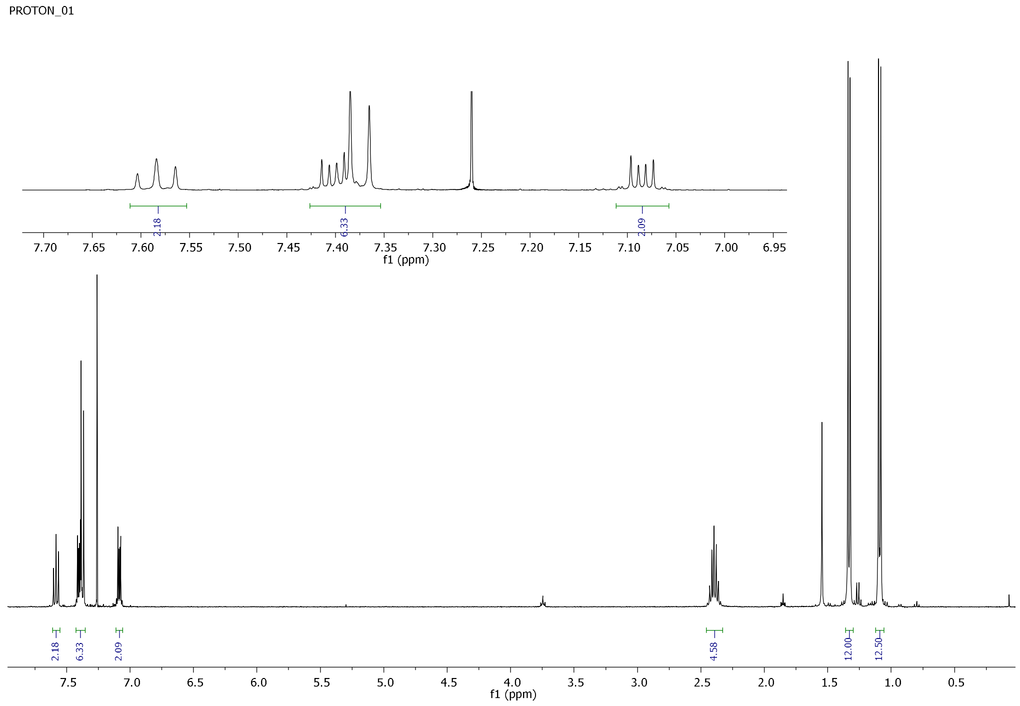

Figure S20: ^{1}H NMR spectrum of **(BZI)AuCl** in $CDCl_3$

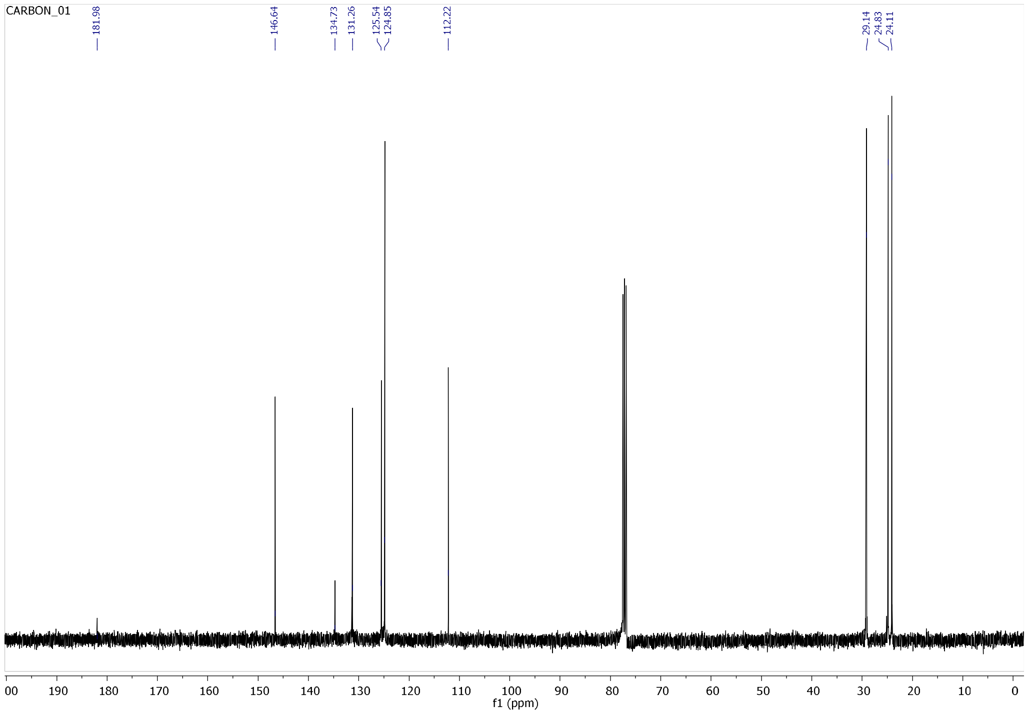

Figure S21: ^{13}C NMR spectrum of **(BZI)AuCl** in $CDCl_3$

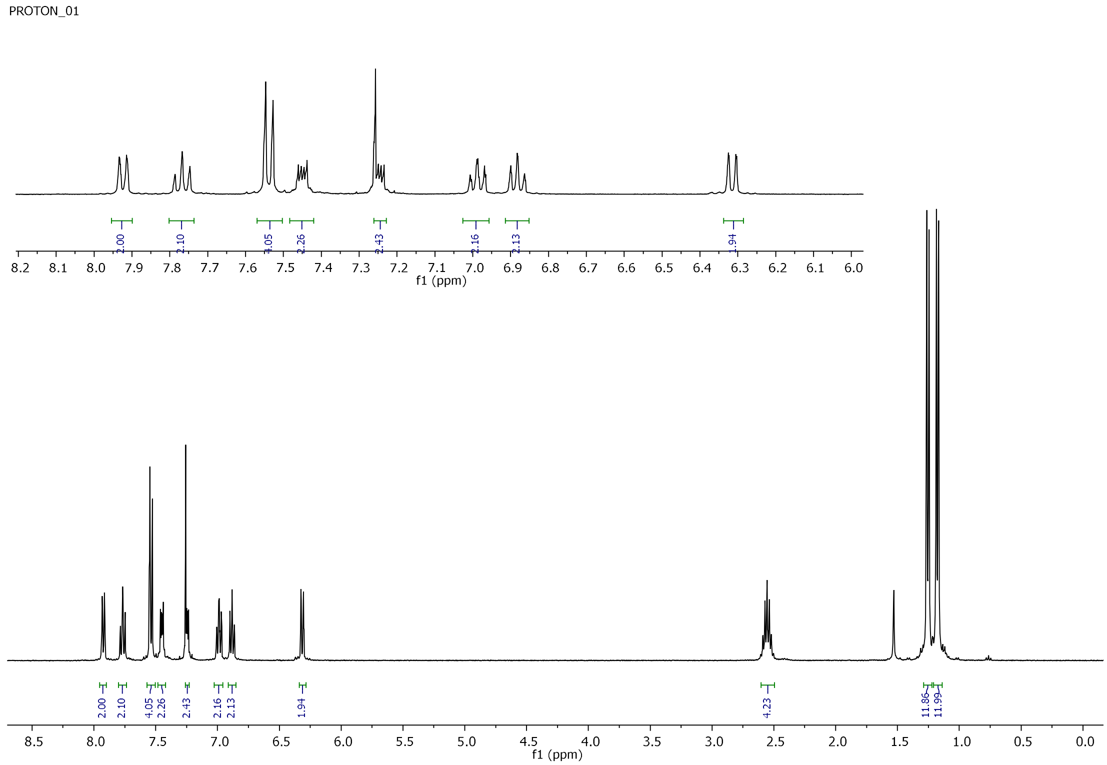

Figure S22: ^{1}H NMR spectrum of **Cu^{BZI}** in $CDCl_3$

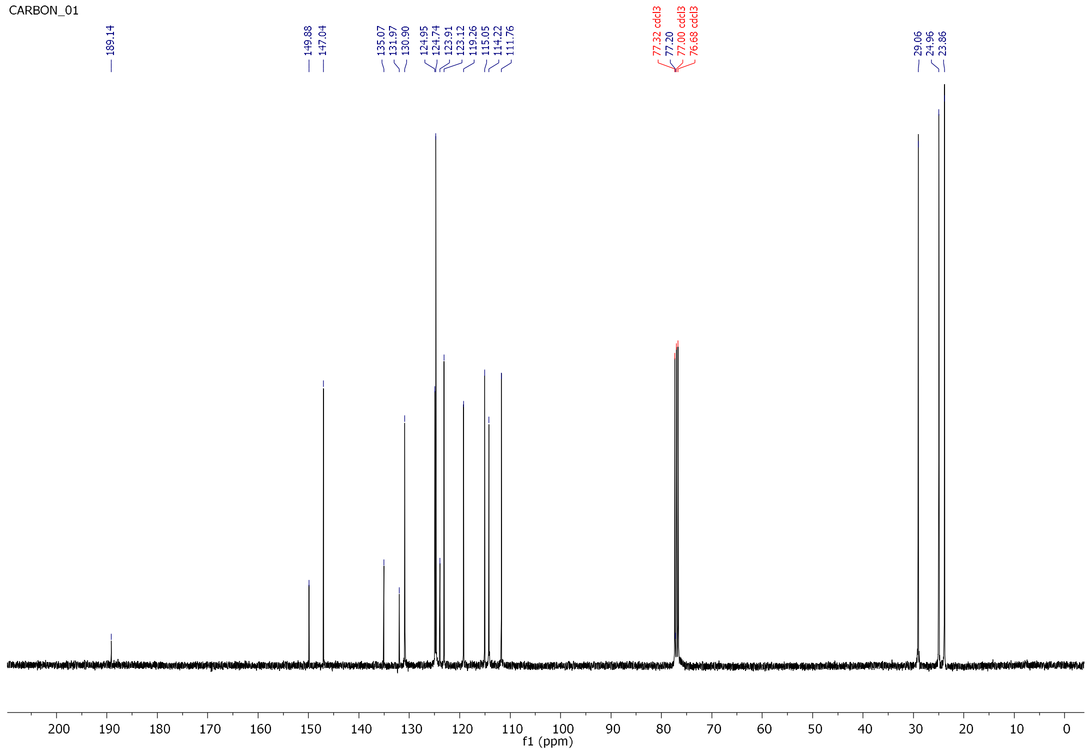

Figure S23: ^{13}C NMR spectrum of **Cu^{BZI}** in $CDCl_3$

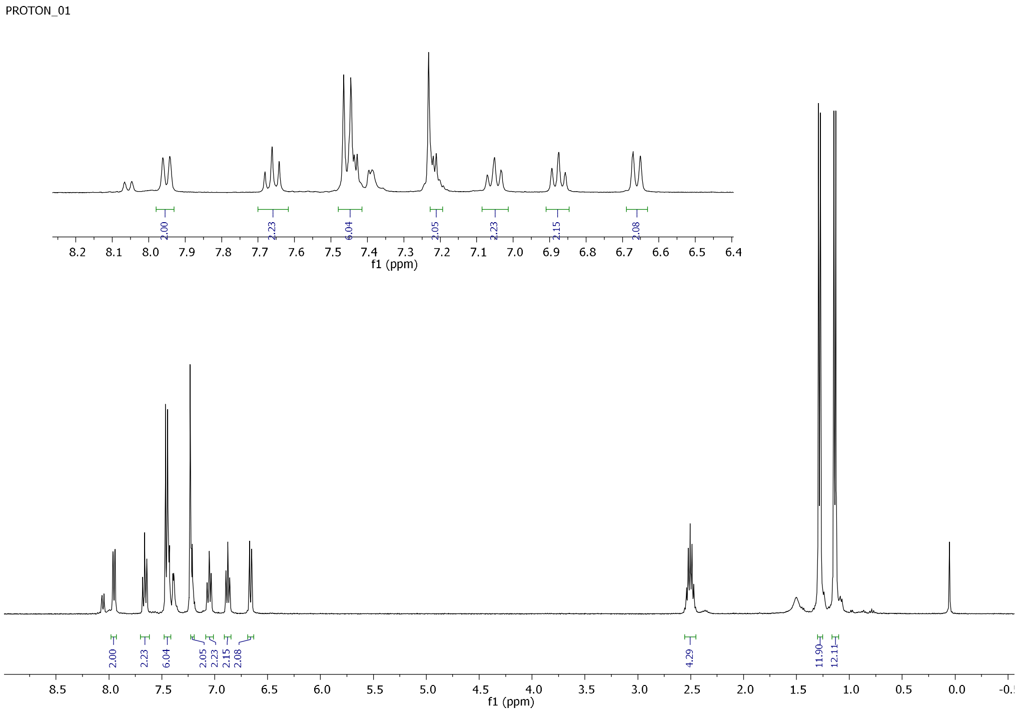

Figure S24: ^{1}H NMR spectrum of **Ag$^{\mathbf{BZI}}$** in $CDCl_3$

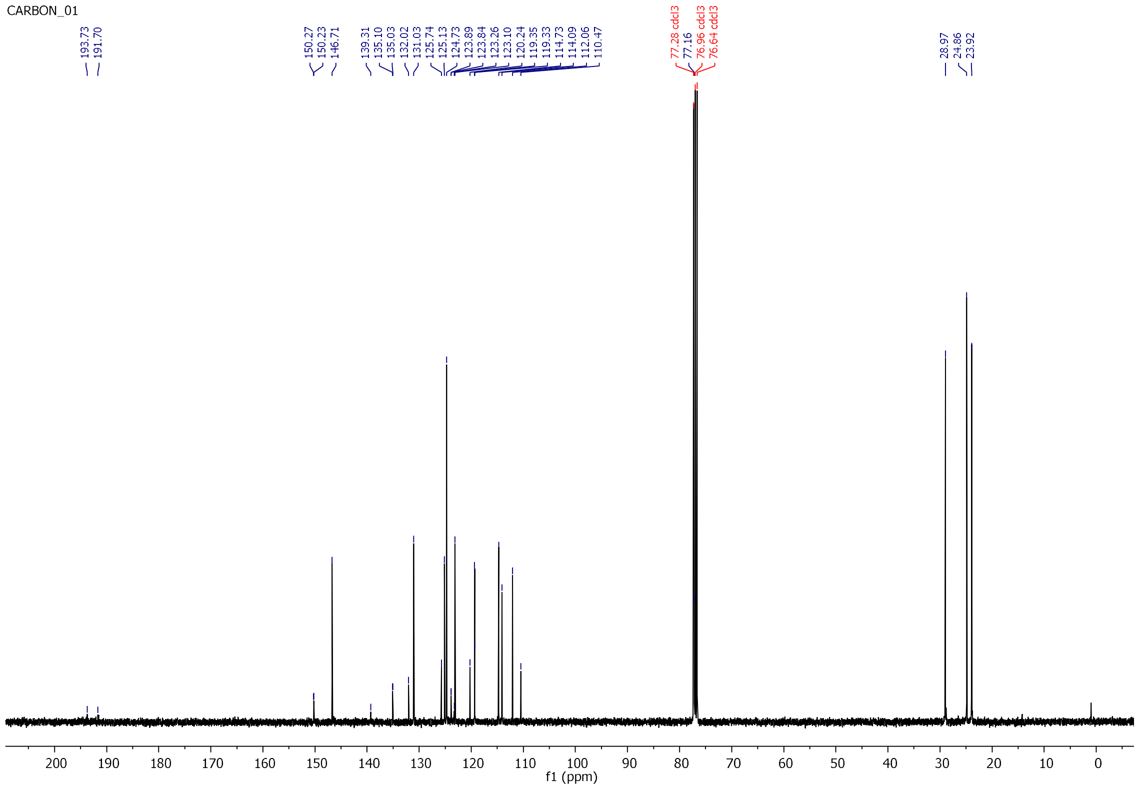

Figure S25: ^{13}C NMR spectrum of **Ag$^{\mathbf{BZI}}$** in $CDCl_3$

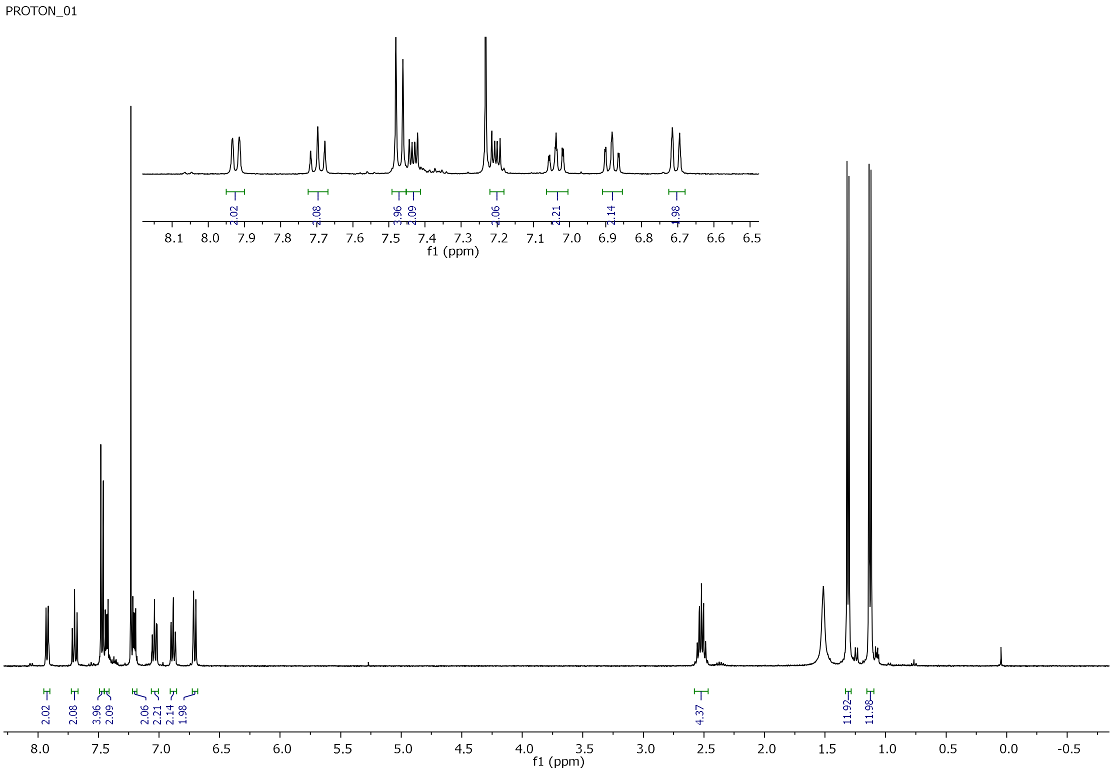

Figure S26: ^{1}H NMR spectrum of **Au$^{\mathbf{BZI}}$** in $CDCl_3$

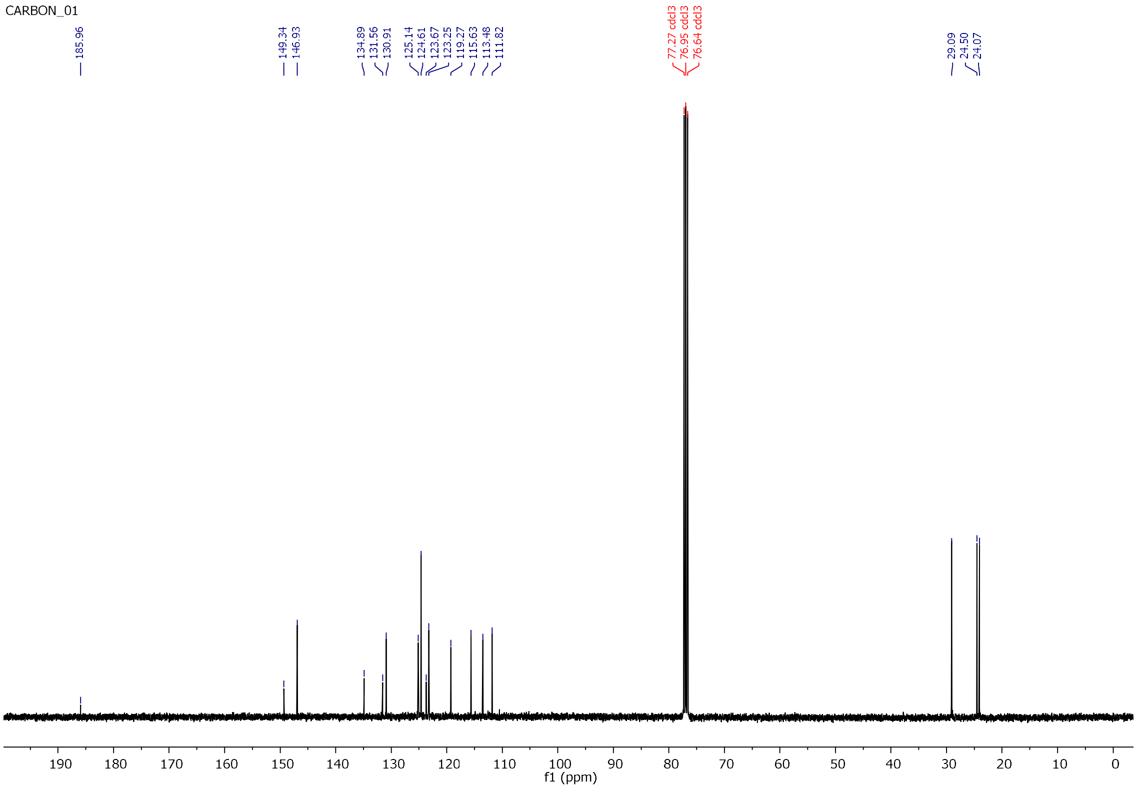

Figure S27: ^{13}C NMR spectrum of **Au$^{\mathbf{BZI}}$** in $CDCl_3$

References

1. Grieco, G.; Blacque, O.; Berke, H., A facile synthetic route to benzimidazolium salts bearing bulky aromatic N-substituents. *Beilstein Journal of Organic Chemistry* **2015,** *11*, 1656-1666.
2. Sworakowski, J.; Lipiński, J.; Janus, K., On the reliability of determination of energies of HOMO and LUMO levels in organic semiconductors from electrochemical measurements. A simple picture based on the electrostatic model. *Organic Electronics* **2016,** *33*, 300-310.
3. Noguchi, Y.; Brutting, W.; Ishii, H., Spontaneous orientation polarization in organic light-emitting diodes. *Japanese Journal of Applied Physics* **2019,** *58*.
4. Altazin, S.; Zufle, S.; Knapp, E.; Kirsch, C.; Schmidt, T. D.; Jager, L.; Noguchi, Y.; Brutting, W.; Ruhstaller, B., Simulation of OLEDs with a polar electron transport layer. *Organic Electronics* **2016,** *39*, 244-249.
5. Jager, L.; Schmidt, T. D.; Brutting, W., Manipulation and control of the interfacial polarization in organic light-emitting diodes by dipolar doping. *Aip Advances* **2016,** *6* (9).
